# The Importance of Evaluating the Lot-to-Lot Batch Consistency of Commercial Multi-Walled Carbon Nanotube Products

**DOI:** 10.3390/nano10101930

**Published:** 2020-09-27

**Authors:** Mai T. Huynh, Jean Francois Veyan, Hong Pham, Raina Rahman, Samad Yousuf, Alexander Brown, Jason Lin, Kenneth J. Balkus, Shashini D. Diwakara, Ronald A. Smaldone, Bryanna LeGrand, Carole Mikoryak, Rockford Draper, Paul Pantano

**Affiliations:** 1 Department of Chemistry and Biochemistry, The University of Texas at Dallas, 800 West Campbell Road, Richardson, TX 75080-3021, USA; Mai.t.Huynh@utdallas.edu (M.T.H.); HongPhuc.Pham@utdallas.edu (H.P.); rxr162230@utdallas.edu (R.R.); Samad.Yousuf@utdallas.edu (S.Y.); Alex.Brown.ut@utdallas.edu (A.B.); jxl140030@utdallas.edu (J.L.); balkus@utdallas.edu (K.J.B.J.); shashinidevika.diwakaramohottalalage@utdallas.edu (S.D.D.); ronald.smaldone@utdallas.edu (R.A.S.); Bryanna.Legrand@utdallas.edu (B.L.); draper@utdallas.edu (R.D.); 2 Department of Materials Science and Engineering, The University of Texas at Dallas, 800 West Campbell Road, Richardson, TX 75080-3021, USA; jfvc@utdallas.edu; 3 Department of Biological Sciences, The University of Texas at Dallas, 800 West Campbell Road, Richardson, TX 75080-3021, USA; mikoryak@utdallas.edu

**Keywords:** nanotoxicity, nanomedicine, engineered nanoparticles, carbon nanomaterials, macrophages

## Abstract

The biological response of multi-walled carbon nanotubes (MWNTs) is related to their physicochemical properties and a thorough MWNT characterization should accompany an assessment of their biological activity, including their potential toxicity. Beyond characterizing the physicochemical properties of MWNTs from different sources or manufacturers, it is also important to characterize different production lots of the same MWNT product from the same vendor (i.e., lot-to-lot batch consistency). Herein, we present a comprehensive physicochemical characterization of two lots of commercial pristine MWNTs (pMWNTs) and carboxylated MWNTs (cMWNTs) used to study the response of mammalian macrophages to MWNTs. There were many similarities between the physicochemical properties of the two lots of cMWNTs and neither significantly diminished the 24-h proliferation of RAW 264.7 macrophages up to the highest concentration tested (200 μg cMWNTs/mL). Conversely, several physicochemical properties of the two lots of pMWNTs were different; notably, the newer lot of pMWNTs displayed less oxidative stability, a higher defect density, and a smaller amount of surface oxygen species relative to the original lot. Furthermore, a 72-h half maximal inhibitory concentration (IC-50) of ~90 µg pMWNTs/mL was determined for RAW 264.7 cells with the new lot of pMWNTs. These results demonstrate that subtle physicochemical differences can lead to significantly dissimilar cellular responses, and that production-lot consistency must be considered when assessing the toxicity of MWNTs.

## 1. Introduction

The unique physicochemical properties of multi-walled carbon nanotubes (MWNTs) lend themselves to a variety of industrial and biomedical applications (for recent reviews, see [1,2,3,4,5,6,7,8,9,10,11,12,13,14,15]). However, there are environmental health and safety (EH&S) concerns associated with MWNTs because of consistent reports that they can be toxic (for recent reviews, see [16,17,18,19,20,21,22]). Moreover, anthropogenic MWNTs have been found in the lungs of asthmatic Parisian children not known to have been exposed to a source of MWNTs, suggesting that MWNTs may be a previously unrecognized air pollutant [23].

MWNTs are synthesized in a range of facilities from small-scale research laboratories to industrial-scale manufacturing plants whose annual production capacities can exceed hundreds of metric tons. Chemical vapor deposition (CVD) processes are the dominant modes of high-volume production because of low energy consumption, low waste generation, and the ability to tailor MWNT properties such as their outer diameters [24]. Even so, commercially available MWNTs with similar product descriptions can have varying physicochemical properties depending on the scale and parameters of the synthetic process, the stringency of quality control measures, and post-production treatments designed to remove contaminants [25,26,27]. For example, MWNTs are frequently purified after synthesis by oxidative treatments to remove metal particles and amorphous carbons, which can generate sp^3^-defect sites and change physicochemical properties based on the exact method and conditions of the post-production treatment used [28]. Unfortunately, the high degree of variability in the physicochemical properties of MWNTs (e.g., composition and impurity content, dimensions and surface topography, crystallinity and types of defects, and agglomeration states to name a few) makes it difficult to assess the EH&S risks of MWNTs and other carbon nanomaterials [29,30,31,32,33,34]. It is therefore not surprising that there are a large number of conflicting reports and knowledge gaps in the nanotoxicity literature concerning MWNTs (for reviews, see [20,35,36,37,38,39,40]). Toward gaining a more thorough understanding of the structure-activity relationships of MWNTs, one mantra that has gained acceptance in the nanotoxicity community is that thorough MWNT characterizations should accompany toxicity investigations, especially since no one analytical technique can probe all aspects of MWNT physicochemical properties that may correlate with a biological response [25,29,31,32,41].

For these reasons, it is not uncommon for researchers to perform extensive material characterizations of commercially obtained MWNTs and to compare their findings with the manufacturer’s product specifications [26,42,43,44,45]. However, beyond the idea of assessing the physicochemical properties of MWNTs from different sources or manufacturers, is the equally important aspect of evaluating different production lots of the same MWNT product (i.e., lot-to-lot batch consistency). While this important aspect has long been endorsed for engineered nanomaterials [32,46,47], to our knowledge, there has only been one report that presents any material characterization data for different production lots of MWNTs; specifically, lots used to prepare MWNT-modified graphite electrodes [48].

Herein, we present a comprehensive physicochemical characterization of two lots of commercial, CVD-synthesized pristine MWNTs (pMWNTs) and carboxylated MWNTs (cMWNTs) used to study the biological responses of mammalian macrophages to MWNTs. There were many similarities between the physicochemical properties of the two lots of cMWNT powders and of bovine serum albumin (BSA)-coated cMWNT suspensions prepared with each cMWNT powder. Equally importantly, BSA-cMWNT suspensions prepared with the two cMWNT powders did not significantly diminish the 24-h proliferation of RAW 264.7 macrophages up to the highest concentration tested (200 μg cMWNTs/mL). The new production lot of cMWNTs is therefore a strong candidate to be a suitable replacement for the original lot of cMWNTs when it is exhausted. Conversely, several physicochemical properties of the two lots of pMWNT powders and of the BSA-coated pMWNT suspensions prepared with each powder were different. The new pMWNTs displayed less oxidative stability, a higher defect density, and a smaller amount of surface oxygen species relative to the original lot of pMWNTs. Also, the concentration of pMWNTs that could be suspended by BSA with the new lot of pMWNTs was significantly lower relative to the original lot of pMWNTs. Most importantly, while the 24-h proliferation of RAW 264.7 macrophages cultured with the original BSA-pMWNT suspensions were statistically similar to the proliferation of cells observed with the two BSA-cMWNT suspensions, the 24-h proliferation of RAW 264.7 cells incubated with BSA-pMWNT suspensions prepared with the new pMWNTs was not. Specifically, the 24-h proliferation of cells incubated with BSA-suspensions of the new pMWNTs at 100 µg/mL was ~20% lower relative to BSA-suspensions of the original pMWNTs at 100 µg/mL, even though the amount of the new pMWNTs accumulated by cells was ~16% less relative to the amount of original pMWNTs accumulated by cells. Furthermore, a 72-h half maximal inhibitory concentration (IC-50) of ~90 µg pMWNTs/mL was determined for RAW 264.7 cells with the new lot of BSA-pMWNTs, making the 2018-pMWNTs significantly more toxic than the original pMWNT lot. These results demonstrate that subtle physicochemical differences can have a significant effect on the response of biological cells to a MWNT product, and that production-lot consistency must be considered when assessing the toxicity or biomedical performance of MWNTs.

## 2. Materials and Methods

### 2.1. Nanomaterials, Chemicals, and Solutions

CVD-synthesized pMWNTs and cMWNTs were purchased directly from the manufacturer. The original pMWNT and cMWNT powders were acquired in 2015 and a second purchase of the exact same products was obtained in 2018; herein, they are denoted as 2015-pMWNTs, 2015-cMWNTs, 2018-pMWNTs, and 2018-cMWNTs. All MWNTs were stored at room temperature in the dark. Caution, a fine-particulate respirator and other appropriate personal protective equipment should be worn when handling dry MWNT powders. Dulbecco’s modified Eagle medium (DMEM) was purchased from Gibco (Grand Island, NY, USA), fetal bovine serum (FBS) was purchased from Atlanta Biologicals (Flowery Branch, GA, USA), and BSA, penicillin (10,000 U/mL), streptomycin (10 mg/mL), and all other chemicals were purchased from Millipore Sigma (Burlington, MA, USA); all chemicals were used as-received. Deionized water (18.3 MΩ-cm) was obtained using a Milli-Q^®^ Integral water purification system (Billerica, MA, USA). Phosphate buffered saline (PBS; 0.8 mM phosphate, 150 mM NaCl, pH 7.4) was sterilized by autoclaving at 121 °C for 45 min. Stock solutions of 100 mg/mL BSA were prepared by dissolving 10 g of BSA in 100 mL of deionized water and adjusting the pH to 7.4. Working solutions of 0.10 mg/mL BSA were prepared by diluting stock BSA solutions with aqueous 10 mM HEPES and filtering the solutions through a 0.22-μm pore membrane; stock and working solutions of BSA were stored at 4 °C in the dark.

### 2.2. CHN/O Analyses

The elemental content of each MWNT powder was determined according to a previously described combustion analysis technique with the exception that all samples were vacuum dried for 4 h at 100 °C prior to analysis [49]. The CHN/O analyses were performed by Micro-Analysis, Inc. (Wilmington, DE, USA) using a Perkin Elmer 2400 Series II CHN/O Analyzer. The CHN analyses were based on the Pregl-Dumas technique using a furnace temperature of 1100 °C in a 100% oxygen atmosphere. The results were reported as the percent by weight of each element with a precision of ±0.30% and a limit of detection (LOD) of <0.10%. The oxygen analysis was based on the Unterzaucher technique using a pyrolysis furnace temperature of 1100 °C and an atmosphere of 95% helium and 5% hydrogen. The results for oxygen were reported as the percent by weight with a precision of ±0.30% and a LOD of <0.10%.

### 2.3. Preparation of BSA-MWNT Suspensions

The sonication and centrifugation protocol described in our previous works [50,51] was used with slight modifications to prepare purified BSA-coated MWNT suspensions. First, 10.0 mg of pMWNT or cMWNT powder was weighed into a pre-cleaned 20-mL glass vial and baked at 200 °C for 2 h to inactivate potential endotoxin contaminants [52]. Next, 10 mL of a 0.10 mg/mL BSA working solution was added to the vial and the mixture was sonicated. Specifically, a single vial was secured in a hanging rack and sonicated for 240 min using an ultrasonic bath sonicator (Elmasonic P30H; Elma Ultrasonic, Singen, Germany) that was operated at 120 W and 37 kHz in a 4 °C cold room. During sonication, the temperature of the bath water was maintained below 18 °C by using a refrigerated water bath circulator (Isotemp 1006S). After sonication, the solution was divided by transferring 1-mL aliquots into ten 1.5-mL centrifuge tubes. One of the 1-mL aliquots of each non-centrifuged BSA-pMWNT or BSA-cMWNT suspension was set aside as the standard suspension, and the MWNT concentrations in these standards were determined by measuring the absorbance at 500 nm using a BioTek Synergy Mx plate reader (Winooski, VT, USA). Next, each standard was serially diluted with a 0.10 mg/mL-BSA working solution to construct pMWNT or cMWNT calibration curves. The remaining nine aliquots were centrifuged at 20,000 RCF for 5 min at 4 °C using an Eppendorf 5417R centrifuge to remove heavier metal-containing MWNTs and bundles, as demonstrated in our previous work [53,54]. The top 900 µL from each supernatant was collected without disturbing the pellet and combined in a sterile vial to afford ~9 mL of a purified BSA-pMWNT or BSA-cMWNT suspension. The concentration of MWNTs in each purified suspension pool was determined using the measured absorbance at 500 nm and the calibration curves described above. Purified BSA-MWNT suspensions were stored at 4 °C in the dark.

### 2.4. Characterization of MWNT Suspensions

Dynamic light scattering (DLS) and zeta potential analyses were used as part of a quality control routine for the preparation of all MWNT suspensions, as previously described [50,51,54]. The particle size distributions, in terms of hydrodynamic diameter, of the BSA-MWNT suspensions were determined by DLS. In brief, aliquots of purified pMWNT or cMWNT suspensions were diluted 1:10 in a 0.10 mg/mL BSA working solution and analyzed with a Zetasizer Nano-ZS 3600 (Malvern Instruments, Worcestershire, UK) using a 633-nm laser and a backscatter measurement angle of 173°. The instrument was calibrated with Polybead^®^ standards (Polysciences, Warrington, PA, USA) and ten consecutive 30-s runs were taken per measurement at 25 °C. The hydrodynamic diameter was calculated using a viscosity and refractive index of 0.8872 cP and 1.330, respectively for deionized water, and an absorption and refractive index of 0.010 and 1.891, respectively, for MWNTs. Zeta potential values were also determined for purified BSA-coated MWNT suspensions that were diluted 1:10 with deionized water. In addition, DLS and zeta potential analyses were performed periodically on purified MWNT suspensions stored at 4 °C. Typically, MWNT suspensions were stable in storage for months, indicated by the lack of aggregates detected by DLS and constant zeta potential results.

### 2.5. Cell Culture

Abelson murine leukemia-virus transformed RAW 264.7 macrophages were purchased from the American Type Culture Collection (ATCC^®^ TIB-71™; Manassas, VA, USA). RAW 264.7 cells were grown in DMEM supplemented with 1.5 mg/mL sodium bicarbonate, 10 mM HEPES (pH 7.4), and 10% (v/v) FBS; the standard incubation conditions were 37 °C in a 5% CO_2_ and 95% air environment.

### 2.6. Crystal Violet Cell Proliferation Assay

Purified BSA-MWNT suspensions were first diluted with a freshly prepared 0.10 mg/mL-BSA working solution to a concentration twice the desired MWNT concentration to be tested. The diluted MWNT suspensions were then mixed 1:1 in equal volumes with 2X-concentrated medium that contained 3.0 mg/mL sodium bicarbonate, 20 mM HEPES (pH 7.4), 20% (*v/v*) FBS, 200 units/mL penicillin, and 0.2 mg/mL streptomycin. The result is a test medium with the same concentration of 10 mM HEPES and 10% FBS as the control medium. ~3.5 × 10^4^ RAW 264.7 cells/well were seeded in 48-well plates and incubated at 37 °C overnight before the medium was replaced with freshly prepared control medium or test medium containing MWNTs at a specified concentration, and incubated for 24 or 72 h. At the end of the incubation, cells were washed three times with fresh medium, two times with PBS, air-dried, and fixed with 4% (*w/v*) paraformaldehyde in PBS. Cell proliferation was determined using a BioTek Synergy Mx plate reader and the standardized crystal violet assay as detailed in our previous work, where it was demonstrated that MWNTs do not interfere with the assay [55]. The dose-response cell proliferation assay with Co^2+^ was identical to the 24-h procedure described above except that CoCl_2_ was first diluted with a freshly prepared 0.10 mg/mL-BSA working solution to a concentration twice the desired CoCl_2_ concentration to be tested. Statistical analyses were performed using a Student’s *t*-test where *p* < 0.05 was considered statistically significant.

### 2.7. Accumulation of pMWNTs by Cells

The following procedure was used to detect the accumulation of pMWNTs by RAW 264.7 cells at 37 °C for 24 h. BSA-pMWNT suspensions were first diluted in a freshly prepared 0.10 mg/mL BSA working solution to twice the desired final MWNT concentrations specified in the experiment. The diluted BSA-pMWNT samples were then mixed 1:1 with 2X-concentrated medium. Total of ~3.5 × 10^5^ cells/well were seeded in 6-well plates and incubated in medium at 37 °C overnight to allow the cells to adhere to the plates. The medium was removed the next day and 2 mL of the appropriate freshly prepared control medium that contained no MWNTs, or test medium that contained a 100 µg/mL pMWNT suspension, was added to each well. Cells were incubated in control or test medium at 37 °C for 24 h. At the end of the incubation, the control and test media were removed by aspiration and the cells were washed three times with fresh medium followed by two washes with PBS. Cells were then lifted off the well using 0.5 mL Accumax^TM^, transferred to a centrifuge tube, and the well was rinsed with 1.5 mL PBS that was subsequently added to the tube to make a final cell suspension of 2 mL/well/tube. Three aliquots of cell suspension, 100 µL each, were used to determine cell counts in each sample using a Beckman Coulter particle counter (Miami, FL, USA) and the cells in the remaining 1.7-mL cell suspension were collected by centrifugation at 1000× *g* for 5 min at 4 °C. The cells in the pellet were lysed in 200 µL of cell lysis buffer that contained 0.25 M Tris-HCl (pH 6.8), 8% (*w/v*) sodium dodecyl sulfate (SDS), and 20% (*v/v*) 2-mercaptoethanol. To ensure complete lysis of the cells, the lysate samples were heated in a boiling water bath for 2 h and then stored at 4 °C. The amounts of pMWNTs in the cell lysate samples were determined using a previously established SDS-polyacrylamide gel electrophoresis (SDS-PAGE) method [56], described next.

### 2.8. Quantitation of pMWNTs Extracted from Cell Lysates by SDS-PAGE

The SDS-PAGE method with optical detection, recently validated by a large-area Raman scan technique [51], was used for quantifying pMWNTs extracted from RAW 264.7 cells. In brief, aliquots of known amounts of pMWNT standard suspensions, lysates of control cells, and lysates of cells treated with BSA-pMWNTs were mixed with 5% 2-mercaptoethanol, 10% glycerol, 62.5 mM Tris-HCl, and 2X-concentrated SDS sample loading buffer to a final concentration of 2% SDS, and boiled for 3 min. Samples at various dilutions and volumes were subsequently loaded into the wells of a SDS-polyacrylamide gel composed of a 4% stacking gel on top of a 10% resolving gel. An electric current was applied at a constant 100 V for 2 h. MWNTs in standard suspensions and in the lysates bind SDS in the sample loading buffer to become negatively charged, and migrate toward the anode upon electrophoresis. The large aspect ratio of MWNTs prevents them from sieving through the pores of a 4% polyacrylamide gel mesh, thus, the MWNTs accumulate at the bottom of the sample loading well during electrophoresis and form a sharp dark band. Following electrophoresis, optical images of the gels were obtained using a flatbed scanner (HP Scanjet G3110), and the pixel intensity of each dark band was quantified using *ImageJ* software. The known amount of pMWNTs in the standards and their corresponding pixel intensities form a linear calibration curve that was used to determine the unknown amount of pMWNTs in cell lysates, based on the pixel intensities of lysate bands loaded in the same gel as the standards. The resultant femtograms of cell-associated pMWNTs/cell were statistically analyzed using a Student’s t-Test where *p* < 0.05 was considered statistically significant.

### 2.9. Inductively Coupled Plasma-Mass Spectrometry (ICP-MS)

All ICP-MS analyses were performed by Precilab, Inc. (Carrollton, TX, USA) using our previously reported acid digestion protocol [49,53]. In brief, a solution of 300 µL of 37% HCl and 100 µL of 69% HNO_3_ was added to ~3.1 mg of a MWNT powder (or to 25.0 mL of a BSA-pMWNT suspension) and bath sonicated for 20 min. Next, the sample was diluted with a 2% HNO_3_ blank solution to a total volume of 50.0 mL and was allowed to settle for 2 h. All metals were calibrated using blanks and standards of 0.050 ppb, 0.100 ppb, 0.250 ppb, and 0.500 ppb concentrations of the respective metals prepared from 1000 ppm standard solutions (Inorganic Ventures); the internal standard was rhodium 103. The samples and standard solutions were aspirated through a nebulizer into a torch chamber and then injected into the plasma through argon gas flow. The determination of Al, Ca, Co, Cr, Fe, Li, Mg, Mn, Ni, K, and Na was performed using a Thermo-Fisher iCap RQ ICP mass spectrometer in cool mode with a 600 W plasma energy. The determination of Ag, As, Au, B, Ba, Be, Bi, Cd, Cu, Ga, Ge, Mo, Nb, Pb, Pt, Sb, Sn, Sr, Ta, Ti, Tl, V, W, Zn, and Zr was performed using a Thermo-Fisher iCap Qs ICP mass spectrometer in hot mode with a 1550 W plasma energy and a kinetic energy discrimination collision cell to remove the chloride interference for As and V. Values are reported in ppm as the mean of n = 2 independent sub-samples and analyses.

### 2.10. Transmission Electron Microscopy (TEM)

TEM was performed using a JEOL JEM 1400 Plus transmission electron microscope (JEOL USA Inc, Peabody, MA, USA) operated at 120 kV with a lanthanum hexaboride filament as the electron source. Each MWNT powder was individually suspended in methanol by bath sonication and an aliquot of the MWNT suspension was deposited on a 300-mesh Cu lacey carbon grid (>70 images were analyzed). High-resolution TEM (HR-TEM) was performed using a JEOL JEM 2100F transmission electron microscope (JEOL USA Inc, Peabody, MA, USA) operated at 200 kV with a field-emission gun as the electron source. Each MWNT powder was individually suspended in ethanol by bath sonication and an aliquot of the MWNT suspension was deposited on a 300-mesh Cu lacey carbon grid (>220 images were analyzed). In all cases, minimized apertures and exposure times were applied to ensure that MWNTs were not damaged by the electron beam. The inside and outer diameters of MWNTs were reported as the mean ± the sample standard deviation (SD).

### 2.11. Thermogravimetric Analyses (TGA)

All TGA measurements were performed using a TA Instruments Q600 thermogravimetric analyzer (TA Instruments, Newcastle, DE, USA) using methods detailed previously [53]. In brief, ~6-mg sub-samples of a thoroughly mixed MWNT powder were transferred into the pan of the analyzer and heated from 25 °C to 800 °C or 1100 °C at 5 °C/min in ultra-pure air (~20.0% O_2_/~80.0% N_2_) at a flow rate of 50 mL/min. A baseline was generated for each scan and buoyancy-corrected, baseline-subtracted thermograms were converted to weight percent. Thermal oxidation temperatures were identified by the peaks from the derivatives of weight percent curves.

### 2.12. Microprobe Raman Spectroscopy

Raman spectra were acquired using a Jobin Yvon Horiba HR 800 high-resolution LabRam Raman microscope (HORIBA Jobin Yvon Inc, Edison, NJ, USA) system equipped with a 250-μm entrance slit and 1100-μm pinhole as described previously [57]. The 633-nm laser excitation was provided by a Spectra-Physics model 127 helium-neon laser operating at 20 mW. A 50×/0.5 NA LM-Plan objective was used with neutral density filter of 1.0. Spectral acquisition was performed with a 1.0-s integration time, a spectral window minimum overlap of 50, and a 3-subpixel average, each spectrum was presented as an average of three scans. Wavenumber calibration was performed using the 520.5 cm^−1^ line of a crystalline silicon wafer and the spectral resolution was ~1 cm^−1^. A 100-μL aliquot of each type of MWNT suspension was deposited on to a crystalline silicon wafer and dried at room temperature overnight; spectra were acquired from at least seven different regions of dried material across the wafer.

### 2.13. Brunauer-Emmett-Teller (BET) Surface Area Measurements

Low-pressure gas adsorption experiments (up to 760 Torr) were carried out on a Micromeritics ASAP 2020 surface area analyzer (Micromeritics Instruments Corp., Norcross, GA, USA), as described previously [58]. All samples were degassed prior to analyses and specific surface areas were determined by a multi-point BET measurement with ultra-high purity nitrogen gas as the adsorbate and liquid nitrogen as the cryogen.

### 2.14. X-ray Diffraction (XRD)

XRD patterns were acquired at room temperature using a Rigaku Ultima IV powder X-ray diffractometer (Rigaku Americas Corporation, The Woodlands, TX, USA) equipped with a Cu Kα vacuum tube and Ni filter as previously described [59]. Individual samples of MWNT powders were placed on a zero-background Si holder (Rigaku) and measured from 2θ = 20–60° with step sizes of 0.02° and a scan rate of 0.5°/min. The Scherrer equation was used to estimate the mean crystallite size (L_C_) from the C(002) Bragg reflection.

### 2.15. X-ray Photoelectron Spectroscopy (XPS)

XPS analysis was performed with a Physical Electronics VersaProbe II surface analysis instrument (Physical Electronics, Chanhassen, MN, USA) equipped with an Al Kα monochromatic X-ray source, as described previously [60]. The base pressure was 4 × 10^−8^ Pa and the electron beam power was set at 50 W (under a potential difference of 15 kV) for an irradiated area at the sample surface of 200 × 200 μm^2^. MWNT powders were deposited onto a gold wafer. The photoelectrons were analyzed with a pass energy of 23.5 eV and an energy step increment of 0.2 eV/step (0.8 eV/step for survey scans). The angle between the sample surface normal and the detector (take-off angle) was set at 45° and the angle between the detector and the X-ray beam was 59°. The charge was referenced to elemental carbon at 284.8 eV. Each spectrum was plotted as an average of twenty scans.

### 2.16. Fourier Transform Infrared (FTIR) Spectroscopy

FTIR spectroscopy was performed using a nitrogen-purged, modified Thermo Nicolet 6700 infrared spectrometer (Thermo Electron Corp., Madison, WI, USA) equipped with a liquid nitrogen-cooled, broadband mercury cadmium telluride (MCT-B) detector [61]. KBr powders were first ground into ultra-fine powders and then heated overnight at 120 °C. Each MWNT powder was mixed uniformly with KBr at a ratio of 1:800, and the mixture was pressed into a pellet using a manual pellet press. Pellets were mounted into a Specac Inc. P/NH 5850c high-pressure cell, and twenty spectra were acquired for each sample and the KBr control with a resolution of 4 cm^−1^.

## 3. Results

### 3.1. CHN/O Analyses of MWNTs

The four MWNT products were reported by the manufacturer to be >95% in purity and the two cMWNT products were reported to comprise ~2% by weight carboxylic acid groups. Lot-acceptance testing was performed using a previously described combustion analysis technique [49]. Table 1 shows that both pMWNT products displayed 96–97% carbon, both cMWNT products displayed ~94% carbon, all products displayed trace amounts of hydrogen, and the 2018 products displayed trace amounts of nitrogen. Overall, the combined carbon, hydrogen, nitrogen, and oxygen contents of the 2015-pMWNTs, 2015-cMWNTs, 2018-pMWNTs, and 2018-cMWNTs powders were 99.52%, 98.18%, 100.26%, and 100.11%, respectively, indicative of MWNT powders that are essentially metal-free. However, while the two 2018 products displayed higher oxygen levels, it should be noted that oxygen determinations carry a higher degree of uncertainty with hygroscopic samples such as MWNTs, which are known to adsorb atmospheric gases and moisture [62]. In a qualitative assessment of dispersibility, both cMWNT products were considered to be relatively hydrophilic because they could be stably suspended in water for >24 h following 1 h of bath sonication at 100 W and 42 kHz without a surfactant. Conversely, both pMWNT products could not be suspended in water following 1 h of sonication without a surfactant (i.e., in each case the majority of pMWNTs would sediment within <2 h). Ultimately, these initial observations, most notably, the high carbon purities, led to the decision to accept each production lot and to move forward with the characterizations of the four BSA-coated MWNT suspensions, followed by cytotoxicity assessments, as described next.

### 3.2. Characterization of BSA-MWNT Suspensions

The sonication and centrifugation protocol developed in our previous works to prepare purified BSA-coated MWNT suspensions [50,51] was modified by using a ten-fold lower concentration of BSA (0.1 mg/mL) in the BSA working solution. Normally, this procedure results in a suspension containing 400–500 μg/mL of BSA-coated MWNTs, which was the case for each MWNT product except for the 2018-pMWNTs (Table 2). Zeta potential and DLS analyses were part of a quality control routine for the preparation of all MWNT suspensions, as previously described [50,51,54]. Table 2 shows that the zeta potentials for the BSA-cMWNTs in deionized water were slightly more negative than those for the BSA-pMWNTs, as expected; and, there were only minor differences in the particle size distributions of BSA-pMWNT and BSA-cMWNT suspensions indicating that the suspended MWNTs possessed similar dimensions with no evidence of major agglomeration. One notable discrepancy, however, was the inability to suspend the 2018-pMWNTs in BSA above a concentration of 275 µg MWNTs/mL (corresponding to a maximum concentration of 136 µg MWNTs/mL when the BSA-MWNT suspension was diluted 1:1 with cell culture medium).

### 3.3. Macrophage Proliferation Assays

**Figure 1:**
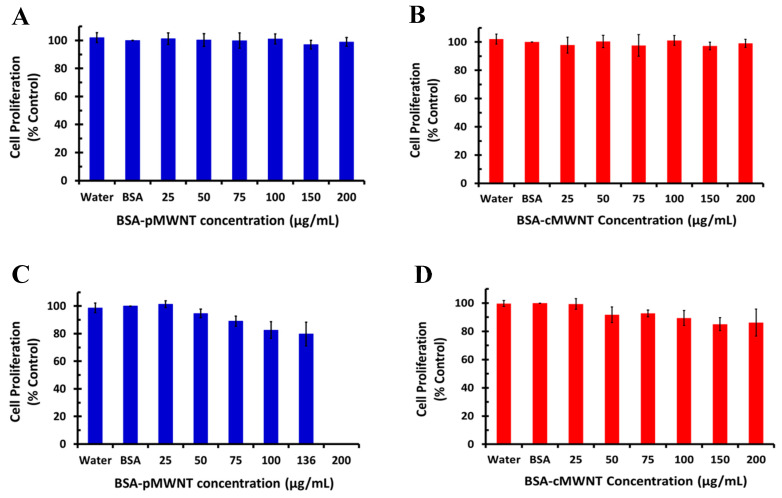
Cell proliferation of RAW 264.7 macrophages cultured with purified BSA-MWNT suspensions prepared with (**A**) 2015-pMWNTs, (**B**) 2015-cMWNTs, (**C**) 2018-pMWNTs, and (**D**) 2018-cMWNTs. MWNTs suspended in a 0.10 mg/mL BSA working solution were mixed with an equal volume of 2X-concentrated medium to produce MWNT concentrations of 0, 25, 50, 75, 100, 150, and 200 µg/mL, except for the 2018-pMWNTs where the highest MWNT concentration that could be made was 136 µg/mL. Exposure to deionized water or a BSA working solution (in the absence of MWNTs) were the controls. Equivalent number of cells were seeded in 48-well plates and incubated at 37 °C under standard cell culture conditions for 24 h prior to the experiment. Cell proliferation after incubation with control and test media for 24 h at 37 °C was determined by the crystal violet assay where the proliferation of control cells exposed to the BSA working solution in the absence of MWNTs was set to 100%. All data sets are the mean of quadruple samples in three independent experiments ± the SD. The 24-h proliferation of RAW 264.7 cells incubated with 2015 MWNTs were essentially identical over the course of ~8 months.

Mammalian macrophages were chosen for this work since they are a key intermediary in nanomaterial pathology and they specialize in phagocytosing foreign particles. The cell proliferation of murine RAW 264.7 macrophages incubated with BSA-MWNT suspensions prepared with each of the four MWNT products was measured after a 24-h exposure to different concentrations of MWNTs using a previously standardized crystal violet assay [55]. Figure 1 shows that there was not a significant decline in the 24-h cell proliferation for RAW 264.7 cells incubated with either the 2015-pMWNTs, the 2015-cMWNTs, or the 2018-cMWNTs at the highest concentration tested (200 µg MWNTs/mL). However, the 24-h proliferation of cells exposed to the 2018-pMWNTs began to decline at 100 µg/mL and the cell count was 78% relative to the control at the highest concentration tested (136 µg pMWNTs/mL). For comparison, the 24-h cell counts for cells incubated with 150 µg/mL BSA suspensions of 2015-pMWNTs, 2015-cMWNTs, and 2018-cMWNTs were 98%, 96%, and 92%, respectively, relative to the control. A direct comparison of the BSA-pMWNT responses at 100 µg/mL revealed that the 24-h proliferation of cells incubated with BSA-suspensions of 2018-pMWNTs was statistically different (*p* = 0.01) relative to BSA-suspensions of 2015-pMWNTs. Interestingly, there were no noticeable differences in the appearance or morphologies of macrophages incubated with BSA-MWNT suspensions prepared with the 2018-pMWNTs relative to the other three MWNT products.

One hypothesis as to why BSA-coated MWNTs prepared with the 2018-pMWNT product decreased the proliferation of RAW 264.7 cells was the possibility that the 2018-pMWNTs adsorbed essential micro-nutrients, or protein growth factors, or both, provided by serum in the cell culture medium, thereby reducing cell proliferation by an indirect mechanism that did not involve a physical nanotube–cell interaction [63,64]. To test this, RAW 264.7 cell proliferation assays were performed in medium containing twice the concentration of serum. For 125-µg/mL BSA-suspensions of 2015-pMWNTs, the 24-h proliferation of cells incubated in 20% FBS (96% relative to the control) was statistically similar to the proliferation of cells incubated in 10% FBS (95% relative to the control); and for 125-µg/mL BSA-suspensions of 2018-pMWNTs, the 24-h proliferation of cells incubated in 20% FBS (82% relative to the control) was also statistically similar to the proliferation of cells incubated in 10% FBS (80% relative to the control; data not shown). These data suggest that the potential depletion of essential serum nutrients from medium by 2018-pMWNTs was not sufficient to generate a false-positive toxicity assessment.

**Figure 2:**
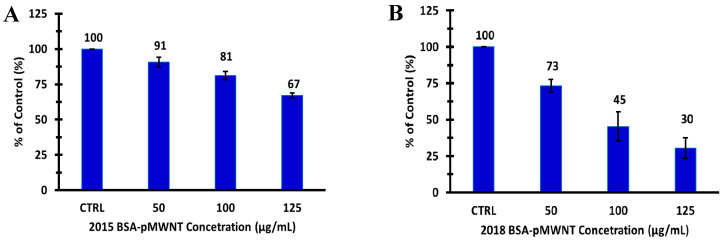
Cell proliferation of RAW 264.7 macrophages cultured with purified BSA-MWNT suspensions prepared with (**A**) 2015-pMWNTs and (**B**) 2018-pMWNTs. MWNTs suspended in a 0.10 mg/mL BSA working solution were mixed with an equal volume of 2X-concentrated medium to produce MWNT concentrations of 0, 50, 100, and 125 µg/mL. Exposure to a BSA working solution (in the absence of MWNTs) was the control. Equivalent number of cells were seeded in 48-well plates and incubated at 37 °C under standard cell culture conditions for 24 h prior to the experiment. Cell proliferation after incubation with control and test media for 72 h at 37 °C was determined by the crystal violet assay where the proliferation of control cells exposed to the BSA working solution in the absence of MWNTs was set to 100%. All data sets are the mean of quadruple samples in three independent experiments ± the SD.

Since the statistical difference from the 24-h cell proliferation assays of the two BSA-pMWNT samples was slight, the response of RAW 264.7 cells as a function of the pMWNT dose was evaluated by extending the pMWNT exposure time to 72 h. While the 72-h proliferation of cells incubated with 125 µg/mL BSA suspensions of 2015-pMWNTs was reduced by ~33% relative to the control, the 72-h proliferation of cells exposed to 125 µg/mL BSA suspensions of 2018-pMWNTs was reduced by ~70% relative to the control, corresponding to an IC-50 of ~90 µg pMWNTs/mL for the 2018-pMWNT product (Figure 2). Moreover, while there were no noticeable differences in the appearance or morphologies of macrophages incubated for 72 h with BSA-MWNT suspensions of 2015-pMWNTs relative to controls, some RAW 264.7 cells exposed to BSA-MWNT suspensions of 2018-pMWNTs for 72 h began to round up in irregular shapes (Figure S1), suggesting that their failure to proliferate was a result of a cytotoxic effect.

### 3.4. Accumulation of pMWNTs by Macrophages 

Another hypothesis as to why BSA-coated MWNTs prepared with the 2018-pMWNT product decreased the proliferation of RAW 264.7 cells was the possibility that the cells phagocytosed more 2018-pMWNTs than 2015-pMWNTs. To investigate this, RAW 264.7 cells were incubated for 24 h in media containing BSA-coated pMWNTs at 100 µg/mL prepared with 2015-pMWNTs or 2018-pMWNTs. After the incubation, the SDS-PAGE method was used to quantify cell-associated MWNTs from cell extracts as previously described [56]. The average amount of 2015-pMWNTs accumulated by cells was 9386 ± 999 fg pMWNTs/cell and that for the 2018-pMWNTs was 7856 ± 350 fg pMWNTs/cell. This data indicate that the difference in the cell proliferation between the two pMWNT products cannot be attributed to a greater amount of 2018-pMWNTs taken-up by RAW 264.7 cells. In summary, the cell accumulation and cell proliferation results were somewhat surprising since the same MWNT products were purchased from the same manufacturer, albeit three years apart; and, because there were no major differences in the carbon purities of the 2018-pMWNT and 2015-pMWNT powders, no major differences were observed in the DLS-determined particle size distributions of BSA-suspensions of 2018-pMWNTs and 2015-pMWNTs. This prompted a more comprehensive physicochemical characterization of all four MWNT products, as described next.

### 3.5. ICP-MS of MWNTs

Since the four MWNT products were synthesized by a Fe/Ni/Co-catalyzed CVD process, ICP-MS analyses were performed to assay for unusually high levels of these metals in the 2018-pMWNT powder whose presence might have affected the proliferation of RAW 264.7 cells. As shown in Table 3, the key findings with the 2015-pMWNT product, which did not alter the 24-h proliferation of the macrophages, were high levels of Ni (~5592 ppm) and Fe (~1690 ppm). In contrast, the unique aspect of the 2018-pMWNT product was a high level of Co (~1242 ppm) relative to the levels found in the 2015-pMWNTs. Thirty-three other metals were also assayed using ICP-MS, and Co was the only element present in the 2018-pMWNT powder at levels that were significantly higher than what was observed in any of the other three MWNT powders (Table S1). However, in this work, it is important to note that MWNT powders were not directly applied to cells, rather BSA-MWNT suspensions were applied and they were purified during preparation by a centrifugation step to remove heavier metal-containing MWNTs and bundles. Therefore, additional ICP-MS analyses of a BSA-pMWNT suspension prepared with the 2018-pMWNT powder were performed. These data revealed ~4 ppm Co, corresponding to a dramatic reduction (>99%) in the Co level relative to the level observed in the MWNT powder, akin to our previous ICP-MS analyses of metals detected in centrifuged carbon nanotube suspensions relative to carbon nanotube powders [53,54]. The literature on the biological effects of Co cations and nanoparticles on mammalian cells was therefore examined. The most relevant finding was reported by Huk and co-workers who studied the effects of 2–10 ppm Co^2+^ ions on J774 mouse macrophages, and who observed significant mortality after a 24-h incubation with 10 ppm Co^2+^ [65]. A dose-response cell proliferation assay with Co^2+^ and RAW 264.7 macrophages was performed next, and a 24-h IC-50 of ~55 ppm Co^2+^ was determined. This result suggests that the 4 ppm Co^2+^ observed in the BSA-pMWNT suspension prepared with the 2018-pMWNT powder did not have a significant acute effect on the proliferation of RAW 264.7 cells.

### 3.6. TEM and HR-TEM Imaging of MWNTs

The inside and outer diameters of MWNTs were estimated from TEM and HR-TEM images. All four MWNT powders were reported by the manufacturer to contain MWNTs with outer diameters of 10–20 nm and inside diameters of 5–10 nm. As shown in Table 4, the observed values closely matched the reported values, and there were no significant differences for the inside and outer diameters among the four MWNT products. Somewhat surprisingly, significant differences between the four MWNT products were not observed via HR-TEM imaging (Figures S2–S10). The majority of MWNTs displayed asymmetric, partially collapsed, open-ends (Figure S2), a small number of MWNTs displayed a relatively symmetric, open-ended nanotube architecture (Figure S6), and very few MWNTs displayed a closed-end nanotube architecture (Figure S4). This is most likely because as-synthesized pMWNTs, and, as-synthesized and oxidized cMWNTs, were reported by the manufacturer to have been shortened by milling to generate the exact lots of pMWNTs and cMWNTs used in this work. HR-TEM images of both pMWNTs and cMWNTs also revealed evidence of hollow tubular cavities (Figures S4 and S10), as well as, asymmetric (bent) sidewalls and symmetric sidewall damage (Figures S2, S3, S5, S7, and S10) as defined by Kónya and co-workers [66]. However, extreme sidewall damage, akin to that observed by Shaffer and co-workers for acid-treated CVD-synthesized MWNTs, was not observed with the two cMWNT products [67]. Additionally, fishbone-type structures (Figure S8), as defined by Su and co-workers [42], and cup-stacked architectures (Figure S9), as defined by Lehman and co-workers [68], were observed frequently for all four MWNT products. Debris-free regions and regions displaying debris of various shapes and sizes, most likely disordered or amorphous carbons akin to that observed by Shaffer and co-workers for both pristine and acid-treated CVD-synthesized MWNTs [67], were observed inside the central cavities and along the sidewalls of all four MWNT products (Figures S5 and S10). However, the extensive degree of sidewall oxidative debris observed by Fairbrother and co-workers for acid-treated MWNTs was not observed with the 2015-cMWNTs and 2018-cMWNTs [69]. Additionally, oxidative debris, defined as carboxylated carbon fragments and polycyclic aromatic hydrocarbons that can be removed by dilute base washings [70], was miniscule for both cMWNT products, as determined by Raman and UV-Vis spectroscopic analyses (data not shown). Finally, metal inclusions, as defined by Andrews and co-workers [71] and Pourchez and co-workers [72], were rarely observed in any of the four MWNT products. Ultimately, the structural morphologies observed for all four MWNT products most closely resembled those presented by Kónya and co-workers who used HR-TEM to study the effects of shortening MWNTs by a ball milling process [66]. It is therefore hypothesized that the similarities observed in the HR-TEM images of the pMWNTs and cMWNTs stemmed primarily from the manufacturer’s milling process, and that the oxidative treatment applied to the cMWNTs did not impart additional structural changes of a significant nature.

### 3.7. TGA of MWNTs

**Figure 3:**
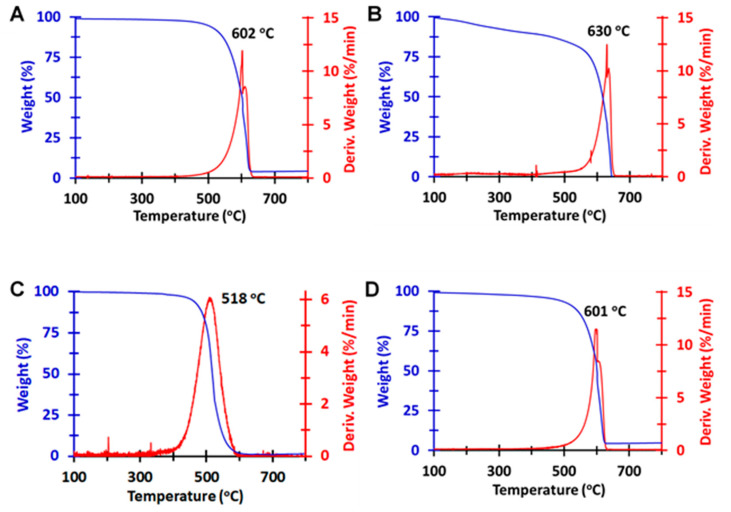
Representative thermograms (in air) showing the weight percent (blue) and derivative of weight percent (red) of the (**A**) 2015-pMWNT, (**B**) 2015-cMWNT, (**C**) 2018-pMWNT, and (**D**) 2018-cMWNT powders.

The TGA weight-percentage and derivative curves of the four MWNT powders are shown in Figure 3. Three of the derivative curves (i.e., the 2015-pMWNTs, the 2015-cMWNTs, and the 2018-cMWNTs) very closely match the profile of the pMWNT derivative curve provided by the manufacturer; specifically, an upward sloping, sharp first peak near 600 °C followed by a closely adjoined, rapidly decaying second peak. The similarities of the main oxidation temperature peaks for these MWNT powders (ranging from 602 °C for the 2015-pMWNTs to 630 °C and 601 °C for the 2015-cMWNTs and 2018-cMWNTs, respectively) is akin to the slight oxidation temperature differences observed by Yim and co-workers for pristine vs. acid-oxidized MWNTs [73]. Oxidation temperatures observed at ~600 °C have also been associated with well-graphitized MWNT structures, which have been reported by Galiotis and co-workers to oxidize between 600 and 700 °C depending on the exact type of MWNT analyzed [74]. In contrast, the derivative curve of the 2018-pMWNTs not only displayed a broader and more Gaussian-like peak profile, the oxidation temperature of this peak occurred ~80 °C earlier than that observed for the 2015-pMWNTs. Defects in carbon nanotube walls are well-known to increase local reactivity, leading to lower oxidation temperatures as observed in TGA mass-loss profiles [75,76]. The main oxidation peak for the 2018-pMWNTs at 518 °C is akin to the oxidation of amorphous or disordered carbons, which typically oxidize at ~500 °C owing to their lower activation energies for oxidation and/or to the presence of reactive defect sites [74,76]. Note, while caution should be exercised in comparing the oxidation temperatures of different MWNTs since these temperatures will vary based on nanotube diameters and ring strain, the similarities in the inside and outer diameters observed via HR-TEM (Table 4) supports an interpretation that the 2018-pMWNTs display less oxidative stability than the 2015-pMWNTs. Moreover, the main oxidation peak for the 2018-pMWNTs at 518 °C very closely matches the Gaussian-like peak profile at ~490 °C observed for 19-nm diameter MWNTs synthesized by a Co-catalyzed CVD process [76], which correlates with the high amount of Co found in the 2018-pMWNT powder by ICP-MS relative to that observed in the 2015-pMWNT powder (Table 3).

In addition to displaying a slight (≤3%) loss of mass at temperatures below 180 °C, corresponding to the release of chemically or physically absorbed gases and moisture [62,74], the next key region of interest in the weight-percentage plots of the four MWNT powders was that between 180 and 450 °C (Figure 3). Weight losses in this region have been attributed to the decomposition of MWNT functional groups, such as surface oxides that evolve CO_2_ and CO gases [62,74,77,78]. As shown in Figure 3A,B for the 2015-MWNT products, weight losses of 1.7% and 9.3% were observed between 180 and 450 °C for the pMWNT and cMWNT powders, respectively, suggesting that the cMWNTs possess more surface oxides than the pMWNTs, as would be expected. As shown in Figure 3C,D for the 2018-MWNT products, a weight loss of 1.9% was observed between 180 and 350 °C for the pMWNT powder and a weight loss of 3.0% was observed between 180 and 450 °C for the cMWNT powder. In summary, it is noteworthy that both pMWNT products displayed only a small amount of surface oxides, which supports the observation that they could not be stably suspended in water following 1 h of sonication. Additionally, it is interesting to note that the 2018-cMWNTs did not qualitatively display as much surface oxides as the 2015-cMWNTs. Finally, while it could be surmised that the 2015-pMWNTs might have been used to generate 2015-cMWNTs, it is safe to conclude that the manufacturer did not use the 2018-pMWNTs to generate 2018-cMWNTs.

### 3.8. Raman Spectroscopy of MWNTs

**Figure 4:**
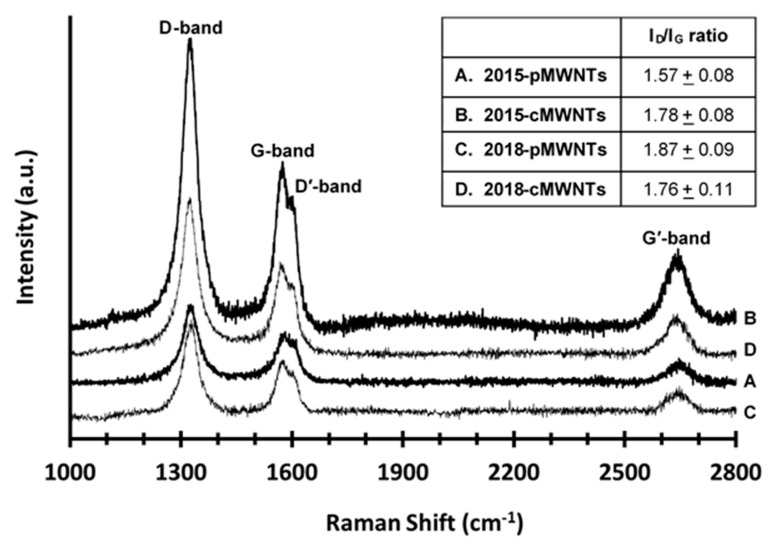
Representative baseline-corrected Raman spectra (632.8-nm laser excitation) of the (**A**) 2015-pMWNT, (**B**) 2015-cMWNT, (**C**) 2018-pMWNT, and (**D**) 2018-cMWNT powders showing characteristic carbon nanomaterial Raman bands (e.g., D-bands at ~1328 cm^−1^, G-bands at ~1577 cm^−1^, D′-bands at ~1604 cm^−1^, and G′-bands at ~2652 cm^−1^). The spectra were offset for clarity. **Inset:** Mean I_D_/I_G_ ratios + SDs of n ≥ 7 analyzed regions for each powder.

The Raman spectra of the four MWNT powders are shown in Figure 4. Each displayed characteristic carbon nanomaterial Raman bands such as the disorder-induced D-band at ~1328 cm^−1^, the tangential graphitic G-band at ~1577 cm^−1^, the disorder-induced G2- or D′-band at ~1604 cm^−1^, and the second-order 2D- or G′-band at ~2652 cm^−1^ [79,80]. The D- and D′-bands are attributed to lattice defects, finite graphene sheets inside carbon nanotube walls, and amorphous or disordered carbons, the G-band is representative of ideal sp^2^-bonded carbon structures, and the G′-band infers long range order in carbon-based structures [42,45]. The mean intensity ratio of the D- and G-bands (I_D_/I_G_) of the four MWNT powders is shown in the Figure 4 inset. This intensity ratio has long been used as a qualitative metric of purity and quality for CVD-synthesized MWNTs of similar diameters with a decrease in the ratio being an indicator of less defect sites (i.e., higher crystallinity) and less amorphous carbon in the sample [69,81]. The I_D_/I_G_ ratio of the 2015-pMWNTs and 2015-cMWNTs (1.57 and 1.78, respectively) follows the expected trend for MWNTs following an oxidative treatment. Specifically, Cui and co-workers and Gogotsi and co-workers both reported I_D_/I_G_-ratio increases of similar magnitudes for CVD-synthesized MWNTs following acid oxidization [82,83]. However, while the 1.78-I_D_/I_G_ ratio of the 2018-cMWNTs was identical to that observed for the 2015-cMWNTs, the 1.87-I_D_/I_G_ ratio for the 2018-pMWNTs was the highest of the four MWNT products. This suggests an increase in defect density and a lower degree of crystallinity of graphitic structures for the 2018-pMWNTs, which is consistent with the lower oxidative stability observed in its TGA profile relative to the three other MWNT products (Figure 3).

### 3.9. XRD Analyses of MWNTs

XRD was performed to analyze the crystallinity of the four MWNT powders. As shown in Figure S11, the main features in the powder X-ray diffraction patterns were peaks located near the (002), (100), and (004) reflections of graphite. Specifically, the intense diffraction peaks at 2θ ≈ 26° can be attributed to the (002) reflection of graphite, the asymmetric diffraction peaks at 2θ ≈ 43° can be assigned to the (100) reflection of graphite, and the high-order diffraction peaks at 2θ ≈ 53° can be assigned to the (004) reflection of graphite that are typically observed with MWNTs [62,71]. For the 2015 products, the (002) reflections for the pMWNTs and cMWNTs were observed at 2θ = 25.98° and 26.02°, respectively, while the (002) reflections for the 2018-pMWNTs and 2018-cMWNTs were observed at 2θ = 25.86° and 25.92°, respectively. In both cases, the (002) reflections for the cMWNTs were shifted by 0.04–0.06°, which was similar to the 2θ-shift of 0.05° observed by Mohanapriya et al. for the (002) reflection of pMWNTs following an oxidative treatment with nitric acid [84]. The (002) reflections were also used to determine the average coherence length (L_C_), the mean crystalline size along the c-axis perpendicular to the long MWNT axis, through the use of the Scherrer equation. The calculated L_C_ values for the two pMWNTs were 9.7 nm and those for the two cMWNTs were 8.8 nm, similar to the 1-nm decrease in L_C_ values observed by Malikov et al. for pMWNTs following an oxidative treatment with nitric acid [85]. Since these values represent an average stacking height of graphitic planes in MWNT walls, a decrease in the L_C_ value for pMWNTs following an oxidative treatment can be attributed to the partial loss of the outermost graphitic layers and the introduction of defects, which reduces the symmetry of the plane [28]. Finally, for all four MWNT powders, there is also a weak reflection observed at 2θ ≈ 44.7° (denoted by the asterisk in Figure S11), which is slightly more pronounced in the XRD pattern of the 2018-pMWNTs. This broad feature could be an amalgamation of the (101) reflection of graphite (2θ ≈ 44.4°), the (111) reflection of Co F*m*$\bar{3}$*m* (2θ ≈ 44.6°), and/or a reflection from other metal-based structures [86,87].

### 3.10. BET Surface Area Measurements of MWNTs

A number of physicochemical characteristics of MWNTs are known to influence BET-determined specific surface areas (SSAs); for example, the number of nanotube walls, nanotube diameters, nanotube bundling, the fraction of open nanotubes, surface functionalization with hydroxyl and carboxyl groups, and types and amounts of metal and amorphous carbon impurities [26,42]. Consequently, there are numerous forewarnings with respect to the use of the BET SSA method with carbon nanotubes because reported SSAs of similar materials frequently differ, and measured SSAs are not always congruent with product specifications [26,68]. Nonetheless, increases in BET-SSAs for acid-oxidized CVD-synthesized MWNTs relative to their pristine counterparts are the norm regardless of the exact oxidant(s) and oxidation reaction conditions employed. This is because oxidative treatments are known to create cavities by opening nanotube ends and by damaging/distorting MWNT sidewalls in the process of removing metal catalysts and amorphous carbon impurities, resulting in an increase in the measured BET-SSA [26,62,70,82]. Additionally, oxidative treatments have been shown to increase BET-SSAs through the generation of functional groups that de-bundle MWNTs by disrupting π–π interactions between pristine nanotube surfaces [26]. Herein, the BET-determined SSAs of the 2015-pMWNT and 2015-cMWNT powders were 91 and 145 m^2^/g, respectively, corresponding to a SSA-increase of ~60%; and, the BET-determined SSAs of the 2018-pMWNT and 2018-cMWNT powders were 191 and 286 m^2^/g, respectively, corresponding to a SSA-increase of ~50%. While the exact reaction conditions of the oxidative treatment performed by the manufacturer are not known, the increases in SSAs measured by the BET measurement for the 2015- and 2018-MWNT product pairs are consistent with the 11–62% increases in BET-SSAs measured by others who evaluated CVD-synthesized pMWNTs and cMWNTs [26,42,70,82,88]. However, it is noteworthy that both of the 2018 products had significantly higher BET-SSAs than the 2015 products.

### 3.11. XPS Analyses of MWNTs

XPS is a method that can be used to determine the elemental composition of a MWNT surface by measuring the binding energy of photoelectrons ejected when the MWNTs are irradiated with X-rays; it is a surface sensitive technique because the escape depth of the photoelectrons amounts to only a few atomic layers [69,89]. Table 5 shows the XPS elemental analyses of the pMWNT and cMWNT powders determined from the C1s and O1s spectra shown in Figures S12 and S13, respectively. The percentages of carbon and oxygen determined by the high-spatial resolution XPS method were consistent with the elemental analysis results shown in Table 1 that were obtained using a bulk method of analysis, except for the lower amount of surface oxygen detected by XPS for the 2018-pMWNTs. As expected, the XPS-determined oxygen-to-carbon ratios of both cMWNT products were greater than their corresponding pMWNT products (Table 5), akin to the increases in XPS-determined oxygen-to-carbon ratios reported by a number of groups who studied the effects of various oxidation reactions on pMWNTs [73,82,90,91].

XPS can be further applied to determine the chemical or electronic state of elements. For example, analysis of the C1s spectra of the four MWNTs shown in Figure S12 indicates that the predominant features at ~284.2 eV correspond to carbons in the sp^2^ hybridization state [73,92]. These peaks resemble the C1s peak of graphite that is typically observed at 284.6 eV, further noting that it is common to see a negative shift of 0.3 eV in the binding energy of MWNTs owing to weaker C–C bonding due to the curvature of graphene sheets and larger interlayer spacings [93]. The presence of functional groups and other defects of MWNTs will influence the full width at half maximum (FWHM) of the sp^2^-hybridized carbon C1s peak [92]. The FWHM values of the graphitic C1s peaks at ~284.2 eV observed for the four MWNTs were all relatively narrow, ranging from 0.9–1.1 eV (Figure S12), and were comparable to 1.2 eV-FWHM values reported for graphite and hydrogen-terminated graphene [92].

Further analysis of the C1s spectra shown in Figure S12 indicates the presence of defects in graphitic structures between 285.1 and 285.7 eV, as well as, satellite peaks between 290.8 and 294.0 eV stemming from π–π* electronic transitions that are representative of disordered sp^2^ carbons [73,91,94]. In addition, there are possibly phenolic, alcohol, and/or ether groups at ~286.5 eV; carbonyl, quinone, carboxyl, and/or lactone groups at ~287.8 eV; and atmospheric contaminants such as O_2_ and carbonates associated with adsorbed CO_2_ between 289–291 eV [91,94]. Unfortunately, the differences in binding energies for these various functional groups are quite small, which is typical for electronegative elements such as oxygen; additionally, discrepancies in the literature regarding the positions of these peaks further contributes to the complexity of the spectral analyses. For example, reported XPS C1s assignments for phenolic and/or alcohol groups on MWNTs span across the range of 285.2–286.8 eV; assignments for ether functional groups on MWNTs span across the range of 286.1–288.0 eV; assignments for carbonyl and/or quinone functional groups on MWNTs span across the range of 286.4–288.1 eV; and assignments for carboxyl and/or lactone functional groups on MWNTs span across the range of 288.0–289.8 eV [69,73,90,91]. While deconvolution of the overlapping peaks is possible, the results of the curve fitting can be ambiguous and will be influenced to some extent by the somewhat arbitrary inputs for the number, shape, and width of the peaks [69,89]. Therefore, the only firm conclusions drawn from these data were that there were no major differences in the C1s spectral profiles of the four MWNTs, except for the slight distinctions with the 2018-pMWNTs in the sp^3^-carbon region and the π–π* region (as denoted by the symbols in Figure S12).

Analysis of the O1s XPS peaks of the four MWNT products revealed notable differences in the spectral profiles. As shown in Figure S13, the O1s peak of the 2015-pMWNTs could be fit well with a single Gaussian peak centered at ~532 eV, while the other MWNTs could not. Instead, the other three MWNT products were best fit with two Gaussian peaks centered at ~531 eV and ~533 eV. Table S2 shows the exact peak positions and the areas under each curve. The O1s spectral profile of the 2015-cMWNTs was broader than that of the 2015-pMWNTs, as expected, and the spectral profile of the 2015-cMWNTs was quite similar to the profile of the 2018-cMWNTs. However, the broader O1s profile of the 2018-pMWNTs did not match that of the 2015-pMWNTs. These data suggest that a variety of surface oxygen functionalities are likely present on the four MWNT products, including but not limited to: (i) physically adsorbed oxygen and/or water, (ii) isolated hydroxyl groups, (iii) carbonyl oxygen atoms in carbonyl, quinone, carboxyl, anhydride, and/or lactone groups, (iv) oxygen atoms from hydroxyl, phenolic, and/or ether groups, and (v) oxygen atoms from carboxylic acids, all of which roughly span the O1s spectral range of 530–535 eV [82,90,91]. Again, it was therefore difficult to distinguish the specific oxygen-containing groups from the O1s spectra with high confidence because unambiguous deconvolution was complicated by the presence of different species with similar and over-lapping binding energies, because of the low amount of oxygen atoms present, and because of discrepancies in the literature regarding the assignments of peak positions [69,95]. Therefore, the only conclusions drawn from these data were that the 2015-cMWNTs, 2018-pMWNTs, and 2018-cMWNTs had slightly different populations of graphitic C–O and C=O species relative to the 2015-pMWNTs (Figure S13 and Table S2).

### 3.12. FTIR Spectroscopy of MWNTs

**Figure 5:**
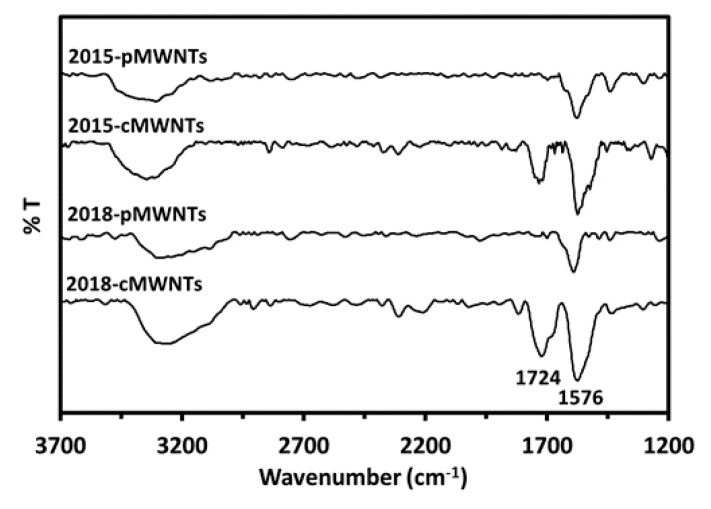
Normalized FTIR spectra of the four MWNT powders; from top to bottom: 2015-pMWNTs, 2015-cMWNTs, 2018-pMWNTs, and 2018-cMWNTs.

FTIR spectroscopy was employed to gain more specific insight into the surface oxygen species present on the four MWNT products. The FTIR spectra shown in Figure 5 display two intense bands for all four MWNTs. The first is the broad band at ~3330 cm^−1^ that is attributed to hydroxyl vibrational stretching modes [υ(O–H)] of surface –O–H groups, –O–H moieties in carboxylic acid groups, water chemisorbed to MWNTs, and/or residual moisture in the KBr pellet [91,96,97]. The second is the band at ~1576 cm^−1^, associated with the carbon skeleton of MWNTs, which is assigned to aromatic carbon–carbon vibrational stretching modes [υ(C=C)] that are polarized by adjacent oxygenated groups [89,91,96,97,98]. It was therefore interesting to note that both of these bands were more intense for the cMWNT products relative to the pMWNTs, as would be expected since it is well-known that treating pMWNTs with oxidizing agents such as the sulfuric acid/permanganate mixture reported by the manufacturer will generate a variety of surface oxygen species, most notably, carboxylic acid and hydroxyl groups. Accordingly, a third band at ~1724 cm^−1^ was also observed only in the spectra of the two cMWNT products, which has been attributed, in general, to the carbonyl vibrational stretching mode [υ(C=O)] of carbonyls and carboxyl groups [91,97], as well as, specifically, to non-conjugated carboxyl carbonyl groups [96,98]. Regardless of this nuance, the overall findings from the FTIR data support the manufacturer’s claim that the cMWNTs were carboxylated. Finally, since milling MWNTs in air can generate surface oxygen functionalities [99], the FTIR data also lend credence to the idea that pMWNTs possess surface hydroxyl groups (likely at defect sites), in part due to the manufacturer’s milling process, and that the milled cMWNTs additionally contain carbonyl groups because only they were treated with oxidizing agents.

## 4. Discussion

### 4.1. Physicochemical Properties of 2018-pMWNTs that Correlate with Reduced Cell Proliferation

A set of pMWNTs and cMWNTs with similar dimensions and purities was purchased in 2015 for evaluating the response of functionalized MWNTs to mammalian macrophages. Lot-acceptance testing was performed using a combustion analysis technique to evaluate the carbon purity of the pMWNTs and cMWNTs. As shown in Table 1, the carbon purity of the 2015 lots of pMWNTs and cMWNTs closely matched the 95% specifications of the manufacturer. Next, purified BSA-coated suspensions of pMWNTs and cMWNTs were prepared for proliferation assays with RAW 264.7 macrophages. As shown in Figure 1A,B, there was not a significant decline in the 24-h proliferation of RAW 264.7 cells with either sample up to the highest concentration tested (200 µg MWNTs/mL). In 2018, as supplies of the 2015 MWNT powders began to run low, a new set of the exact same pMWNT and cMWNT products was purchased. Lot-acceptance testing was performed and both of these 2018 materials closely matched the 95% carbon purity levels stated by the manufacturer (Table 1). Next, purified BSA-suspensions of pMWNTs and cMWNTs were prepared for proliferation assays with RAW 264.7 cells. As shown in Figure 1C,D, while there was not a significant decline in the 24-h proliferation of RAW 264.7 cells with the 2018-cMWNTs up to the highest concentration tested (200 µg MWNTs/mL), the proliferation of RAW 264.7 macrophages decreased to 78% of the control when incubated with 136 µg/mL of the 2018-pMWNTs, the highest concentration of BSA-suspended MWNTs that could be prepared in cell culture medium using the 2018-pMWNT powder. It should also be noted that when freshly prepared samples of 2015 BSA-MWNTs were tested ~8 months apart, the 24-h proliferation of RAW 264.7 cells incubated with 2015 MWNTs were essentially identical, indicating that potential aging of the 2015 MWNT powders was not a source of variability (*vide infra*). Moreover, as shown in Figure 2, a 72-h IC-50 of ~90 µg pMWNTs/mL was determined for BSA-suspensions of 2018-pMWNTs and some RAW 264.7 cells exposed to BSA-MWNT suspensions prepared with the 2018-pMWNTs were rounded after 72 h (Figure S1), consistent with their failure to proliferate being a result of a cytotoxic effect.

Suspensions of all four BSA-coated MWNTs were characterized before the cell proliferation assays were performed, and as shown in Table 2, the DLS-determined dimensions of particles were quite similar, indicating that discrepancies in the agglomeration of MWNTs was not the cause of the biological response observed with the 2018-pMWNTs. It should also be noted that when freshly prepared samples of 2015 BSA-MWNTs were tested ~1 year apart, the relative MWNT concentrations, HDDs, and zeta potential values were essentially identical, indicating that potential aging of the 2015 MWNT powders was not a source of variability (*vide infra*). Additionally, TEM and HR-TEM imaging did not reveal any significant differences in the inside and outside diameters of the four MWNTs (Table 4), and HR-TEM imaging did not reveal any major morphological differences among the four MWNTs (Figures S2–S10). Furthermore, the amounts of 2015-pMWNTs and 2018-pMWNTs taken up by RAW 264.7 cells did not correlate with the 24-h cell proliferation results; in other words, the reduced cell proliferation observed with the 2018-pMWNTs was not because the cells accumulated more 2018-pMWNTs than 2015-pMWNTs. In fact, the accumulated amount of BSA-pMWNTs prepared with the 2018 product was ~16% less than the accumulated amount of BSA-pMWNTs prepared with the 2015 product.

ICP-MS analyses revealed ~50× more Co in the 2018-pMWNT powder relative to the Co levels found in the 2015-pMWNTs (Table 3), and ~4 ppm Co was observed in BSA-pMWNT suspensions prepared with the 2018-pMWNT powder. A dose-response cell proliferation assay with Co^2+^ and RAW 264.7 macrophages yielded a 24-h IC-50 of ~55 ppm Co^2+^, indicating that exposure to 4 ppm Co^2+^ should not have a significant acute effect on the proliferation of RAW 264.7 cells. While Liu and co-workers observed that Co nanoparticles had a more significant effect on RAW 264.7 cells than Co^2+^ ions [100], Co was not observed in the XPS survey scans of any MWNT powder and HR-TEM imaging rarely revealed metal inclusions in any MWNT sample. Therefore, the presence of Co was ruled out as the causation of the reduced proliferation of RAW 264.7 cells incubated with BSA-suspension of 2018-pMWNTs.

A perfect crystalline carbon nanotube comprises only hexagonal rings of sp^2^-hybridized carbons. However, synthesized MWNTs are far from perfect and various amounts and types of defects are generated during MWNT growth and subsequent post-synthetic treatments [30,99]. Defective MWNT structures have been classified into four main groups: topological differences in shape due to ring sizes other than hexagons, sp^3^-hybridized carbon atoms, incomplete bonding defects (e.g., vacancies and dislocations), and doping with elements other than carbon [101]. Both the TGA (Figure 3) and Raman analyses (Figure 4) indicated an increased density of defect sites with the 2018-pMWNTs relative to the 2015-pMWNTs; specifically, the 2018-pMWNTs displayed lower oxidative stability and a higher I_D_/I_G_ ratio.

The percentages of carbon and oxygen determined by XPS were consistent with the elemental analysis results obtained using a combustion analysis technique, except for the lower amount of surface oxygen detected by XPS for the 2018-pMWNTs (Table 1 and Table 5). Analysis of the C1s spectral profiles of the four MWNTs revealed no major differences except for the slight distinctions for the 2018-pMWNTs in the sp^3^-carbon region and the π–π* region (Figure S12), whereas analysis of the O1s spectral profiles revealed that the 2015-cMWNTs, 2018-pMWNTs, and 2018-cMWNTs had slightly different populations of graphitic C–O and C=O species relative to the 2015-pMWNTs (Figure S13 and Table S2). FTIR spectroscopy, however, provided more specific functional group information, namely, that both cMWNT products were indeed functionalized with carbonyl groups whereas the pMWNTs were not (Figure 5). XPS and FTIR spectroscopic analyses were also used to assess whether atmospheric aging had any effect on the physicochemical properties of the 2015 MWNTs. For example, Liu et al. simulated atmospheric aging by studying the oxidation (by O_3_ or OH·) of single-walled carbon nanotubes, and observed increases in surface carboxylic acids or esters (i.e., an enhancement of the O/C ratio), but they did not observe any changes in toxicity with human A549 adenocarcinoma-derived alveolar epithelial cells and THP-1 leukemia-derived peripheral blood monocytes [102]. Herein, all MWNTs were stored in the dark to avoid UV-catalyzed reactions, and increases in the O/C ratios as a function of time were not observed, most notably, with the older 2015 MWNTs. In fact, it was the newer 2018 pMWNTs that possessed the lowest O/C ratio (Table 5), and, as shown by the FTIR spectra in Figure 5, there was no evidence of carboxylic acids in either of the pMWNT materials.

Determining the fundamental origin(s) of a cytotoxic response to a MWNT sample is a complex endeavor because many MWNT physicochemical determinants are interrelated and it is difficult to systematically decouple them [30]; for example, milling MWNTs to modulate defect densities will also shorten MWNTs [99]. MWNT defects are a physicochemical property that have been proposed to affect the toxicity of mammalian cells [72,103,104,105,106,107]. Unfortunately, many toxicity reports focusing on defects were not limited to this single physicochemical parameter, rather, studies involved MWNTs with structural defects additionally had differences in other determinants such as lengths, BET-SSAs, and/or surface functionalization. One compelling in vivo study was reported by Lison and co-workers who progressively and selectively modified MWNTs by grinding and heating pMWNTs to introduce and modify structural defects [108]. Their results with Wistar rats indicated that the presence of MWNT structural defects mediated pulmonary toxicity, and they postulated that the toxic potential of MWNTs could be partially abolished by the elimination of surface defects. While additional well-designed studies to predict toxic responses based on individual physicochemical properties are warranted, the premise that structural defects are a key determinant of toxicity might help to explain the reduced proliferation of RAW 264.7 cells incubated with the 2018-pMWNTs that possessed more defects relative to the 2015-pMWNTs.

A more established tenet is that structural defects, surface chemistry, surface curvature, and the surface area of a MWNT are decisive factors involved in the dynamic formation of a protein corona [62,109]. In the present case, the protein corona is first given a coating of BSA that adsorbs onto MWNTs in the process of preparing BSA-MWNT suspensions, followed by an additional layer of macromolecules, primarily proteins that coat BSA-MWNTs (and compete with BSA for MWNT surface sites) once BSA-MWNTs are mixed with cell culture medium that contains serum. Thus, a protein corona, whose formation is governed in part by MWNT surface properties, can partially screen the intrinsic properties of a MWNT surface, and provide a BSA-MWNT with a new biological identity [30,109]. The compositions of protein coronas formed on different functionalized MWNTs are complex and unique; for example, liquid chromatography-tandem mass spectrometry was used to show that cMWNTs bound a greater overall number of proteins (and different types of proteins) from cell culture medium relative to pMWNTs [108]. This is important because the biological response of cells to MWNTs typically starts with their binding to the plasma membrane, sometimes via a membrane receptor, and consequent internalization inside a vesicle and ultimately into the cell [30,106,109,110].

### 4.2. The Unsuitability of the 2018-pMWNTs as a Replacement for the 2015-pMWNTs

TGA and Raman analyses suggest the 2018-pMWNTs had more defects relative to the 2015-pMWNTs (Figure 3 and Figure 4), and the XPS elemental analysis of the 2018-pMWNTs revealed the lowest surface oxygen levels of the four MWNT products (Table 5). The differences in the surface chemistry and structural defects of the 2018-pMWNTs (relative to the 2015-pMWNTs) could therefore have an effect on the protein corona formed when each BSA-pMWNT suspension was prepared. In fact, the relative concentration of MWNTs observed in BSA-MWNT suspensions prepared with the 2018-pMWNTs was ~34% less than that for the 2015-pMWNT suspension (Table 2). The differences in the surface chemistry and structural defects could also have had an effect on the protein corona formed when each BSA-pMWNT suspension was mixed with the DMEM/FBS cell culture medium and then presented to cells. However, further studies would be required to quantify protein corona differences with these two pMWNT lots and their effects on RAW 264.7 cell proliferation and accumulation. Nonetheless, it is straight-forward to conclude that RAW 264.7 macrophages respond differently to BSA-pMWNT suspensions prepared with the 2018-pMWNT powder, and that the lot of 2018-pMWNTs is not a suitable replacement for the lot of 2015-pMWNTs.

### 4.3. The Suitability of the 2018-cMWNTs as a Replacement for the 2015-cMWNTs

There are many similarities in the physicochemical properties of the 2015-cMWNT and 2018-cMWNT powders. The carbon purities of the 2015-cMWNTs and 2018-cMWNTs determined by combustion analyses (94.30% and 94.19%, respectively) were in close agreement (Table 1), as were the carbon purities (94.37% and 95.57%, respectively) determined by XPS (Table 5). The XPS-determined surface oxygen percentages of the 2015-cMWNTs and 2018-cMWNTs were similar (5.63% and 4.43%, respectively), and the 0.060-oxygen/carbon ratio of the 2015-cMWNTs was only slightly greater than the 0.046-oxygen/carbon ratio of the 2018-cMWNTs (Table 5). The TGA-determined weight loss observed between 180 and 450 °C corresponding to surface oxides was also greater for the 2015-cMWNTs relative to the 2018-cMWNTs (Table 2; 9.3% and 3.0%, respectively). However, the shapes of the predominant TGA peaks of the 2015-cMWNTs and 2018-cMWNTs were quite similar (Figure 3), as were their oxidation temperatures (630 °C and 601 °C, respectively). Substantial differences in the levels of elements determined by ICP-MS were not observed between the 2015-cMWNTs and 2018-cMWNTs (Table S1). The TEM-determined outside diameters of the 2015-cMWNTs and 2018-cMWNTs (19 ± 5 nm and 21.4 ± 4 nm, respectively) and inside diameters (5.7 ± 1.7 nm and 5.6 ± 2.1 nm, respectively) were also comparable (Table 3). Additionally, HR-TEM imaging of the 2015-cMWNTs and 2018-cMWNTs did not reveal any striking differences in morphologies (Figures S6–S10). Somewhat surprisingly, the BET-determined SSA of the 2018-cMWNTs (~286 m^2^/g) was roughly twice that of the 2015-cMWNTs (~144 m^2^/g). However, the Raman spectral profiles and the I_D_/I_G_ ratios of the 2015-cMWNTs and 2018-cMWNTs (1.78 and 1.76, respectively) were highly comparable (Figure 4), as were their XRD patterns (Figure S11) and XPS C1s and O1s spectral profiles (Figures S12 and S13). Finally, both the 2015-cMWNTs and 2018-cMWNTs displayed a carbonyl vibrational stretching mode at ~1724 cm^−1^ in their FTIR spectra supporting the manufacturer’s claim that the cMWNTs were carboxylated (Figure 5).

Suspensions of BSA-coated cMWNTs prepared with the 2015-cMWNT and 2018-cMWNT powders were also quite similar. As shown in Table 2, the relative concentrations of suspended MWNTs (~496 and ~456 μg/mL, respectively), the DLS-determined hydrodynamic diameters (~86 and ~84 nm, respectively), and the zeta potentials (~34 and ~33 mV, respectively) of the two BSA-cMWNT suspensions were quite comparable. Most importantly, Figure 1 shows that the 24-h proliferation of RAW 264.7 macrophages cultured with BSA-cMWNT suspensions prepared with the 2015-cMWNT and 2018-cMWNT powders were statistically similar up to the highest concentration tested (200 μg cMWNTs/mL). Ultimately, while every physicochemical parameter was not identical, the combined results indicate that the 2018 production lot of cMWNTs is a strong candidate as a suitable replacement for the 2015 lot of cMWNTs for the purpose of studying the biological response of mammalian macrophages to functionalized MWNTs.

## 5. Conclusions

A comprehensive physicochemical characterization of two commercial lots of CVD-synthesized pMWNTs and cMWNTs revealed many similarities between the two cMWNT products and several key differences between the two pMWNT products. The 2018-pMWNTs displayed less oxidative stability, a higher defect density, and a smaller amount of surface oxygen species relative to the 2015-pMWNTs. Additionally, the concentration of pMWNTs that could be suspended by BSA with the 2018-pMWNTs was significantly lower relative to the 2015-pMWNTs. Most importantly, while the 24-h proliferation of RAW 264.7 macrophages cultured with BSA-suspensions of 2015-pMWNTs were statistically similar to the proliferation of cells observed with the two BSA-cMWNT suspensions, the 24-h proliferation of RAW 264.7 cells incubated with BSA-suspensions of 2018-pMWNTs was not. Specifically, the 24-h proliferation of cells incubated with BSA-suspensions of 2018-pMWNTs at 100 µg/mL was ~20% lower relative to BSA-suspensions of 2015-pMWNTs at 100 µg/mL, even though the amount of the 2018-pMWNTs accumulated by cells was ~16% less relative to the amount of 2015-pMWNTs accumulated by cells. Furthermore, a 72-h IC-50 of ~90 µg pMWNTs/mL was determined for RAW 264.7 cells with BSA-suspensions of 2018-pMWNTs, making the 2018-pMWNTs significantly more toxic than the 2015-pMWNTs.

The differences in the surface chemistry and structural defects of the 2018-pMWNTs relative to the 2015-pMWNTs likely influenced the protein corona that was formed when BSA-pMWNT suspensions were prepared, which in turn could affect the binding and subsequent accumulation of the 2018-pMWNTs by RAW 264.7 cells. Reactive structural defects, a key determinant of toxicity, also likely influenced the diminished 24-h proliferation of RAW 264.7 cells, as well as, the 72-h toxicity observed with the 2018-pMWNTs. This work therefore demonstrates (i) the difficulty in assessing the role of a single physicochemical property of a MWNT product to an observed biological response, (ii) that subtle physicochemical differences can have a significant effect on the response of biological cells to a MWNT product, and (iii) that production-lot consistency must be considered when assessing the toxicity or biological activity of MWNTs and other carbon nanomaterials.

## Figures and Tables

**Table 1:** CHN/O elemental percentages of the pristine multi-walled carbon nanotube (pMWNT) and carboxylated MWNT (cMWNT) powders.

Element	2015-pMWNTs (%)	2015-cMWNTs (%)	2018-pMWNTs (%)	2018-cMWNTs (%)
**C**	96.49	94.30	97.29	94.19
**H**	1.06	1.08	0.06	0.11
**N**	0.00	0.00	0.01	0.20
**O**	1.97	2.80	2.90	5.60
**Total**	99.52	98.18	100.26	100.11

**Table 2:** Particle size and zeta potential analyses of purified bovine serum albumin (BSA)-MWNT suspensions.

BSA–MWNT Suspension ^1^	Relative MWNT Concentration (μg/mL) ^2^	Dynamic Light Scattering ^3^		Zeta Potential (mV) ^6^
		HDD (nm) ^4^	PDI ^5^	
**2015-pMWNTs**	417 ± 19	81.4 ± 5.4	0.21	−31.8 ± 1.9
**2015-cMWNTs**	496 ± 34	85.7 ± 6.8	0.20	−33.8 ± 1.6
**2018-pMWNTs**	275 ± 18	81.3 ± 2.1	0.20	−29.1 ± 1.8
**2018-cMWNTs**	456 ± 15	84.1 ± 1.6	0.21	−32.8 ± 1.5

^1^ Purified BSA-MWNT suspensions were prepared by a sonication and centrifugation technique. ^2^ Relative MWNT concentrations were measured using the absorbance at 500 nm of each respective suspension; the values are presented as the mean ± the SD of n ≥ 3 independent samples. ^3^ Aliquots of purified pMWNT or cMWNT suspensions were diluted 1:10 in 0.10 mg/mL BSA working solutions. ^4^ Hydrodynamic diameter (HDD); the values are presented as the mean ± the SD of n ≥ 3 independent samples. ^5^ Polydispersity index (PDI). ^6^ Aliquots of purified pMWNT or cMWNT suspensions were diluted 1:10 in deionized water; the values are presented as the mean ± the SD of n ≥ 3 independent samples. The relative MWNT concentrations, HDDs, and zeta potentials of the 2015 MWNTs were essentially identical over the course of ~1 year.

**Table 3:** ICP-MS analyses of pMWNT powders.

Element	2015-pMWNTs	2018-pMWNTs
**Fe (ppm)**	1689.8	475.4
**Ni (ppm)**	5591.6	8.8
**Co (ppm)**	24.6	1241.8

**Table 4:** TEM analyses of pMWNT and cMWNT powders.

MWNT Product	Outer Diameter (nm)	Inner Diameter (nm)
**2015-pMWNTs**	18 ± 3	5.6 ± 1.3
**2015-cMWNTs**	19 ± 5	5.7 ± 1.7
**2018-pMWNTs**	21 ± 4	5.3 ± 0.6
**2018-cMWNTs**	21 ± 4	5.6 ± 2.1

**Table 5:** XPS elemental analyses of pMWNT and cMWNT powders.

MWNT Powder	% Carbon ^1^	% Oxygen ^2^	Subtotal ^3^	O/C ^4^
**2015-pMWNTs**	96.4	3.6	100.0	0.04
**2015-cMWNTs**	94.4	5.6	100.0	0.06
**2018-pMWNTs**	99.1	0.9	100.0	0.01
**2018-cMWNTs**	95.6	4.4	100.0	0.05

^1^ Percentage of atomic carbon determined from the area of the respective C1s peak at ~284 eV, normalized to 100% of the elements detected. ^2^ Percentage of atomic oxygen determined from the respective area of the O1s peak at ~532 eV normalized to 100% of the elements detected. ^3^ All four samples were composed of carbon and oxygen; no other elements were observed in the respective survey scans noting however that XPS cannot detect H or He. ^4^ Ratio of the atomic percentages of oxygen to carbon.

## Supplementary Materials

The following are available online at https://www.mdpi.com/2079-4991/10/10/1930/s1; Figure S1: Phase contrast images of RAW 264.7 cells following 72 h of incubation with BSA-pMWNTs. Table S1: ICP-MS analyses of pMWNTs and cMWNTs. Figures S2–S10: HR-TEM images of pMWNTs and cMWNTs. Figure S11: XRD patterns of pMWNTs and cMWNTs. Figure S12: C1s XPS spectra of pMWNTs and cMWNTs. Figure S13: O1s XPS spectra of pMWNTs and cMWNTs. Table S2: O1s XPS peak analyses from pMWNTs and cMWNTs.
