# Supplementary material for: The Importance of Evaluating the Lot-to-Lot Batch Consistency of Commercial Multi-Walled Carbon Nanotube Products
Source: Nanomaterials (Basel). 2020 Sep 27;10(10):1930. doi: 10.3390/nano10101930 (PMC7601794; doi:10.3390/nano10101930)
Supplement: Supplementary file 1 [file nanomaterials-10-01930-s001.pdf]

**Supplementary Materials for**

# The Importance of Evaluating the Lot-to-Lot Batch Consistency of Commercial Multi-Walled Carbon Nanotube Products

Mai T. Huynh,[1] Jean Francois Veyan,[2] Hong Pham,[1] Raina Rahman,[1] Samad Yousuf,[1] Alexander Brown,[1] Jason Lin,[1] Kenneth J. Balkus, Jr.,[1] Shashini D. Diwakara,[1] Ronald A. Smaldone,[1] Bryanna LeGrand,[1] Carole Mikoryak,[3] Rockford Draper,[1,3] and Paul Pantano[1]*

[1] Department of Chemistry and Biochemistry, The University of Texas at Dallas, 800 West Campbell Road, Richardson, Texas 75080-3021, USA
[2] Department of Materials Science and Engineering, The University of Texas at Dallas, 800 West Campbell Road, Richardson, Texas 75080-3021, USA
[3] Department of Biological Sciences, The University of Texas at Dallas, 800 West Campbell Road, Richardson, Texas 75080-3021, USA

* Correspondence: pantano@utdallas.edu (P.P.)

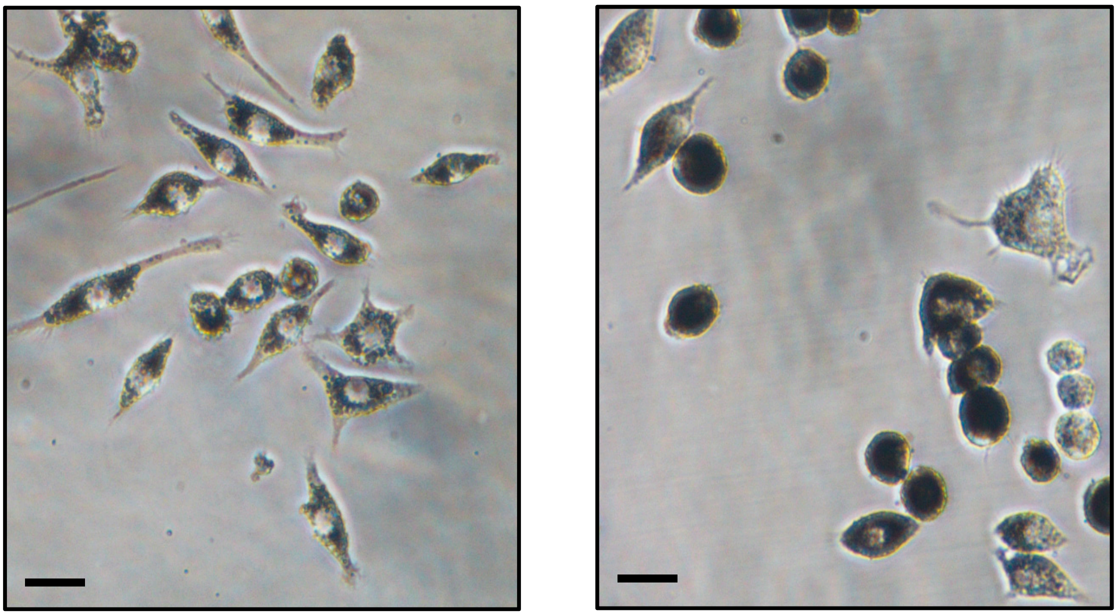

**Figure S1.** Representative phase contrast images acquired using an inverted light microscope (Nikon SMZ745T) equipped with a digital camera (Nikon DS-Fi2) of RAW 264.7 cells following 72 h of incubation with 125-μg/mL BSA-suspensions of 2015-pMWNTs (left) or 2018-pMWNTs (right). The scale bars represent 10 μm.

**Table S1.** ICP-MS analyses of pMWNT and cMWNT powders.

| | | 2015-pMWNTs | 2015-cMWNTs | 2018-pMWNTs | 2018-cMWNTs | MDL* |
|---|---|---|---|---|---|---|
| Aluminum | Al | 35.622 | 7.103 | 11.496 | 7.953 | 0.012 |
| Antimony | Sb | 0.138 | 0.149 | 0.025 | 0.005 | 0.002 |
| Arsenic | As | 0.245 | 0.052 | 0.345 | 0.052 | 0.052 |
| Barium | Ba | 2.991 | 2.954 | 1.562 | 1.159 | 0.001 |
| Beryllium | Be | 0.014 | 0.014 | 0.014 | 0.014 | 0.014 |
| Bismuth | Bi | 0.003 | 0.001 | 0.022 | 0.470 | 0.001 |
| Boron | B | 14.625 | 18.884 | 9.189 | 10.957 | 0.174 |
| Cadmium | Cd | 0.029 | 0.004 | 0.005 | 0.008 | 0.004 |
| Calcium | Ca | 1,186.718 | 1,343.731 | 651.953 | 1,419.302 | 0.063 |
| Chromium | Cr | 8.932 | 0.837 | 3.714 | 18.656 | 0.005 |
| Cobalt | Co | 24.597 | 2.679 | 1,241.834 | 4.183 | 0.014 |
| Copper | Cu | 2.169 | 0.348 | 0.649 | 3.231 | 0.012 |
| Gallium | Ga | 0.026 | 0.005 | 0.015 | 0.012 | 0.005 |
| Germanium | Ge | 0.030 | 0.014 | 0.014 | 0.014 | 0.014 |
| Gold | Au | 0.003 | 0.003 | 0.003 | 0.003 | 0.003 |
| Iron | Fe | 1,689.820 | 28.690 | 475.353 | 123.964 | 0.022 |
| Lead | Pb | 0.543 | 0.033 | 0.076 | 0.721 | 0.005 |
| Lithium | Li | 0.130 | 0.085 | 0.092 | 0.055 | 0.001 |
| Magnesium | Mg | 197.304 | 198.221 | 66.185 | 174.402 | 0.002 |
| Manganese | Mn | 7.283 | 0.308 | 5.841 | 12.433 | 0.007 |
| Molybdenum | Mo | 110.852 | 4.300 | 3.237 | 9.936 | 0.009 |
| Nickel | Ni | 5,591.619 | 78.091 | 8.792 | 97.132 | 0.029 |
| Niobium | Nb | 0.189 | 0.012 | 0.008 | 0.007 | 0.002 |
| Platinum | Pt | 0.008 | 0.050 | 0.048 | 0.008 | 0.008 |
| Potassium | K | 20.245 | 30.789 | 13.715 | 33.888 | 0.021 |
| Silver | Ag | 1.675 | 0.318 | 0.009 | 0.014 | 0.003 |
| Sodium | Na | 78.042 | 72.556 | 31.502 | 49.317 | 0.004 |
| Strontium | Sr | 3.462 | 5.675 | 2.028 | 5.344 | 0.001 |
| Tantalum | Ta | 0.002 | 0.004 | 0.001 | 0.001 | 0.001 |
| Thallium | Tl | 0.001 | 0.001 | 0.001 | 0.001 | 0.001 |
| Tin | Sn | 0.147 | 0.035 | 0.145 | 0.179 | 0.003 |
| Titanium | Ti | 5.707 | 6.533 | 1.246 | 0.804 | 0.011 |
| Tungsten | W | 0.097 | 0.025 | 0.048 | 0.023 | 0.003 |
| Vanadium | V | 0.121 | 0.070 | 0.938 | 0.292 | 0.004 |
| Zinc | Zn | 10.458 | 2.308 | 1.314 | 2.965 | 0.018 |
| Zirconium | Zr | 16.233 | 479.190 | 119.315 | 42.038 | 0.010 |

* MDL = Method detection limit; MWNT data listed in blue font indicates that the observed results were at or below the MDL. All values are reported in units of ppm.

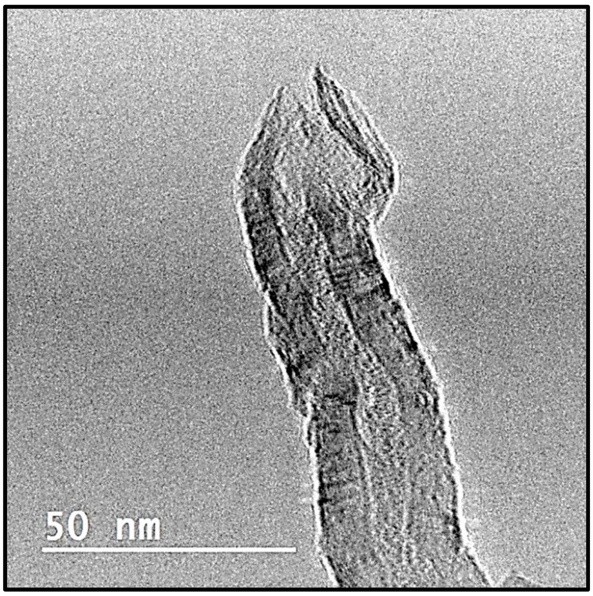


**Figure S2.** Representative HR-TEM image of a 2015-pMWNT highlighting asymmetric (bent) sidewall damage and a partially-collapsed, open-end.

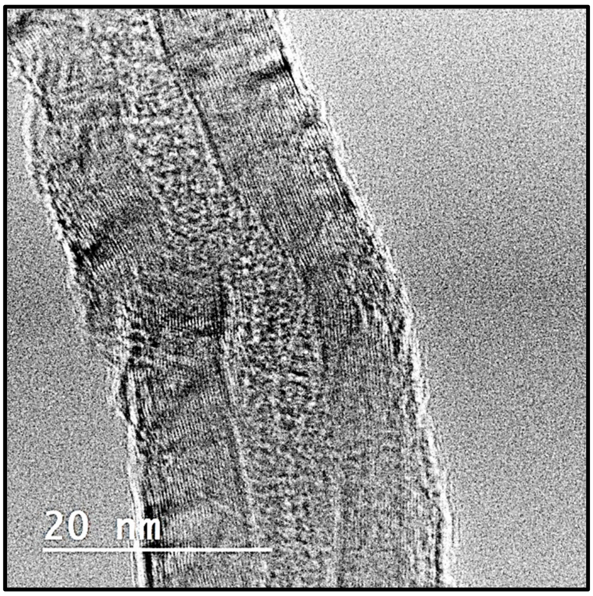


**Figure S3.** Representative HR-TEM image of a 2015-pMWNT showing asymmetric (bent) sidewall damage.

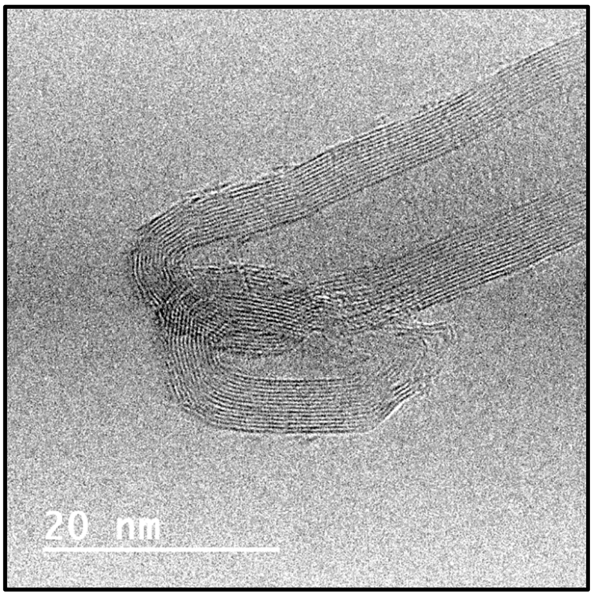


**Figure S4.** Representative HR-TEM image of a 2018-pMWNT highlighting a closed-end nanotube architecture, a hollow inner-cylinder, and a unique anomaly at the tip.

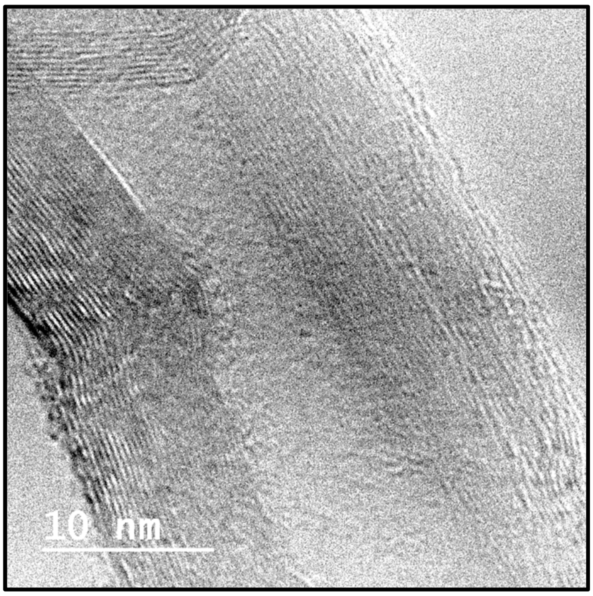


**Figure S5.** Representative HR-TEM image of a 2018-pMWNT highlighting asymmetric (bent) sidewall damage and sidewall debris.

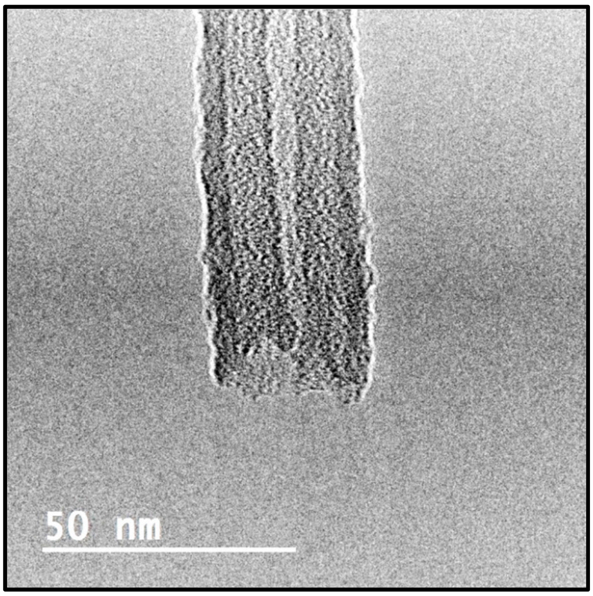


**Figure S6.** Representative HR-TEM image of a 2015-cMWNT highlighting a relatively symmetric, open-end nanotube architecture.

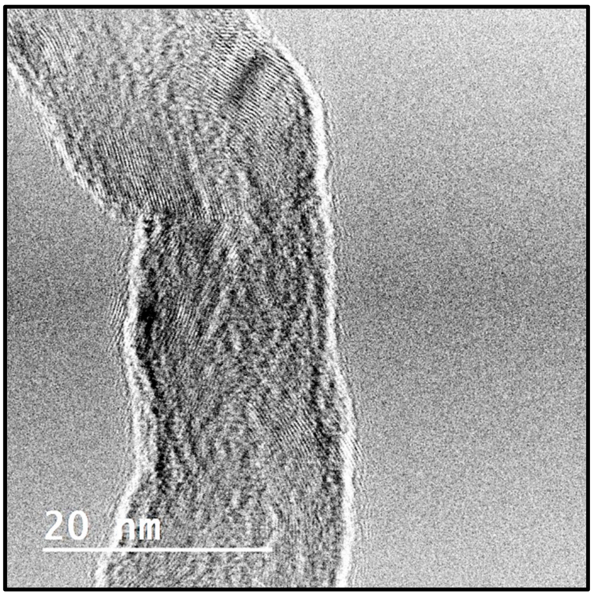


**Figure S7.** Representative HR-TEM image of a 2015-cMWNT highlighting asymmetric (bent) sidewall damage.

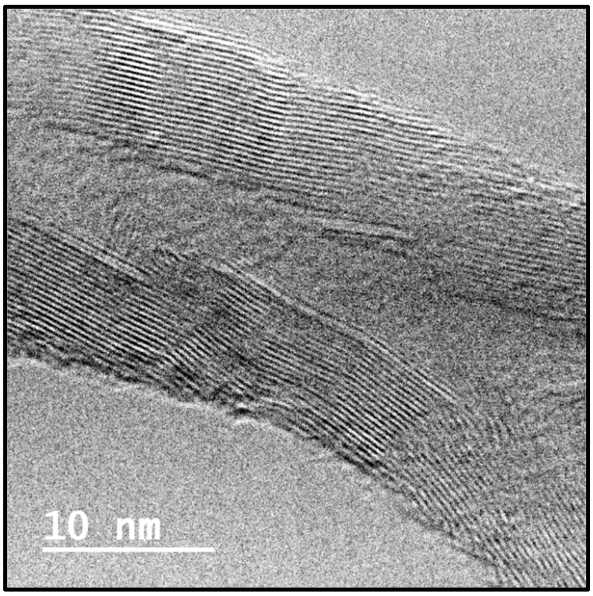


**Figure S8.** Representative HR-TEM image of a 2018-cMWNT highlighting a fishbone-type structure.

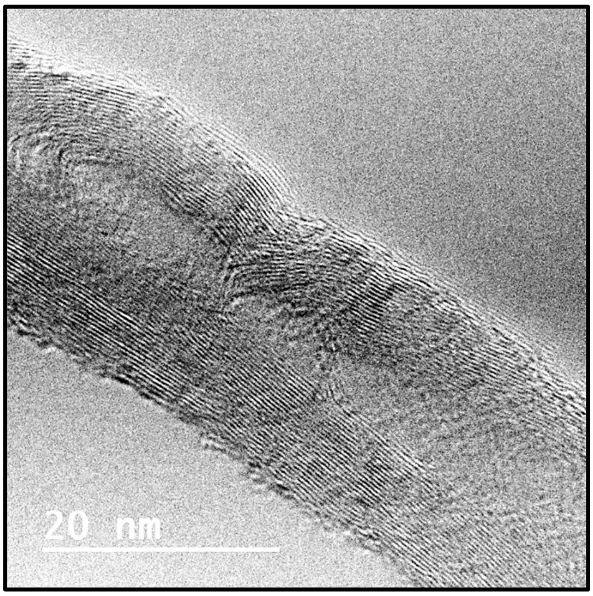


**Figure S9.** Representative HR-TEM image of a 2018-cMWNT highlighting cup-stacked structures.

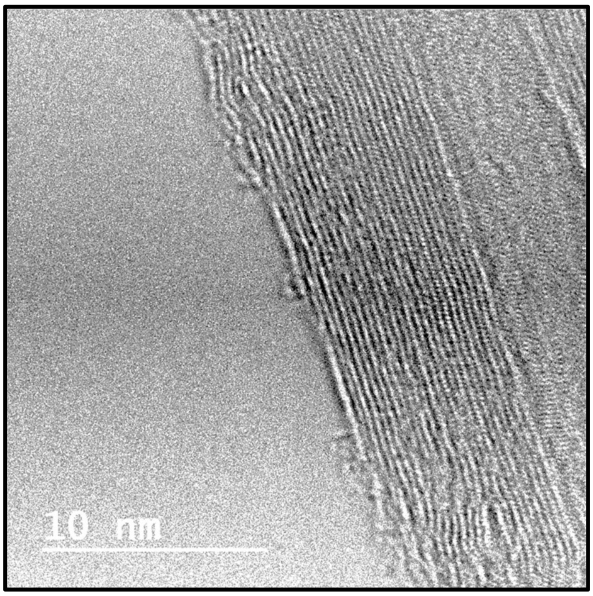


**Figure S10.** Representative HR-TEM image of a 2018-cMWNT highlighting a hollow inner-cylinder, sidewall damage, and sidewall debris.

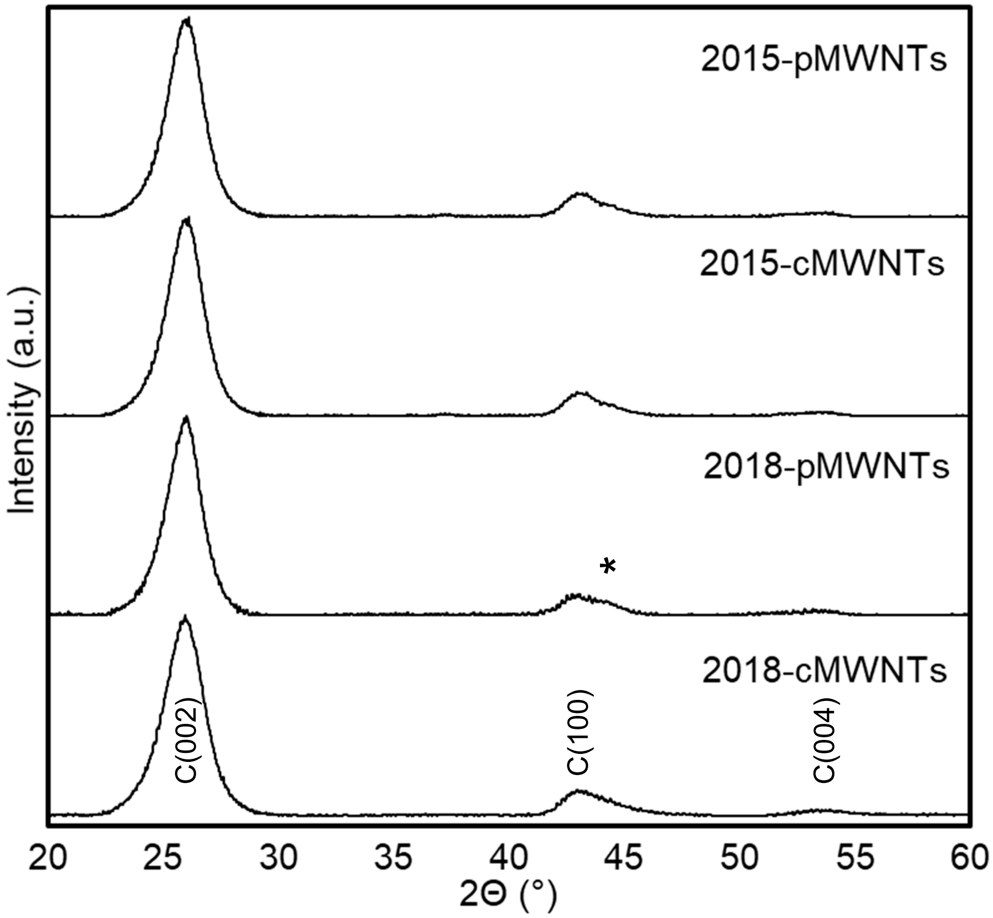


**Figure S11.** Representative XRD patterns (normalized and offset for clarity) of the 2015-pMWNT, 2015-cMWNT, 2018-pMWNT, and 2018-cMWNT powders showing the C(002), C(100), and C(004) diffraction peaks characteristic of an ideal graphite phase.

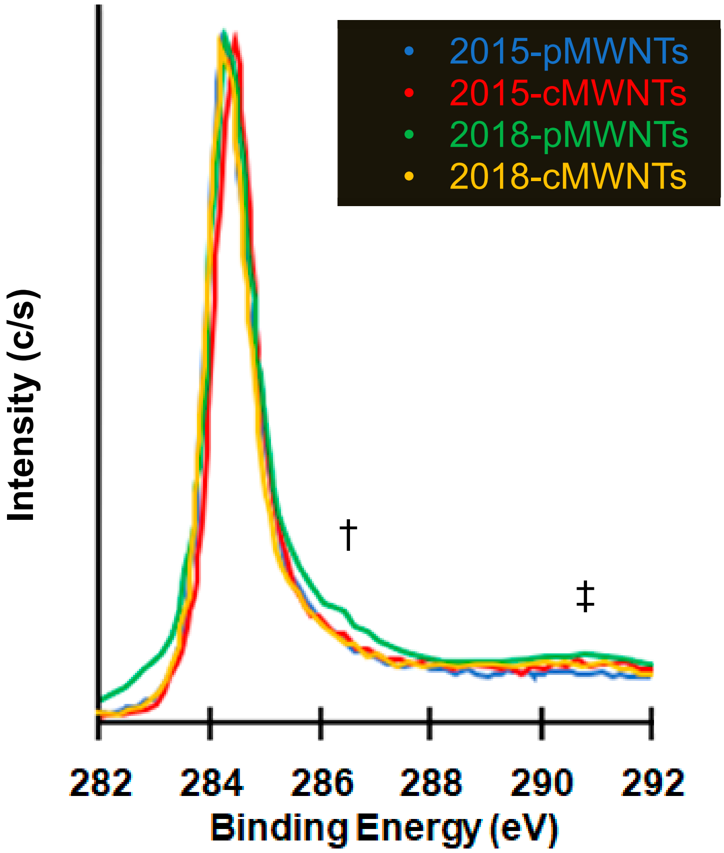


**Figure S12.** Representative C1s XPS spectra of the four MWNT powders where the major peak corresponds to $sp^2$-hybridized carbons is centered: at 284.2 eV (FWHM ≈ 1.1 eV) for the 2015-pMWNTs, at 284.4 eV (FWHM ≈ 1.1 eV) for the 2015-cMWNTs, at 284.2 eV (FWHM ≈ 1.1 eV) for the 2018-pMWNTs, and at 284.3 eV (FWHM ≈ 0.9 eV) for the 2018-cMWNTs. Note, that the <0.2 eV-differences in the positions of the main C1s peaks were considered insignificant based on the instrument's energy resolution. The symbols represent C1s spectral regions associated with $sp^3$-hybridized carbons (†) and the $\pi$–$\pi^*$ electronic transition that is representative of disordered $sp^2$ carbons (‡); see text for details.

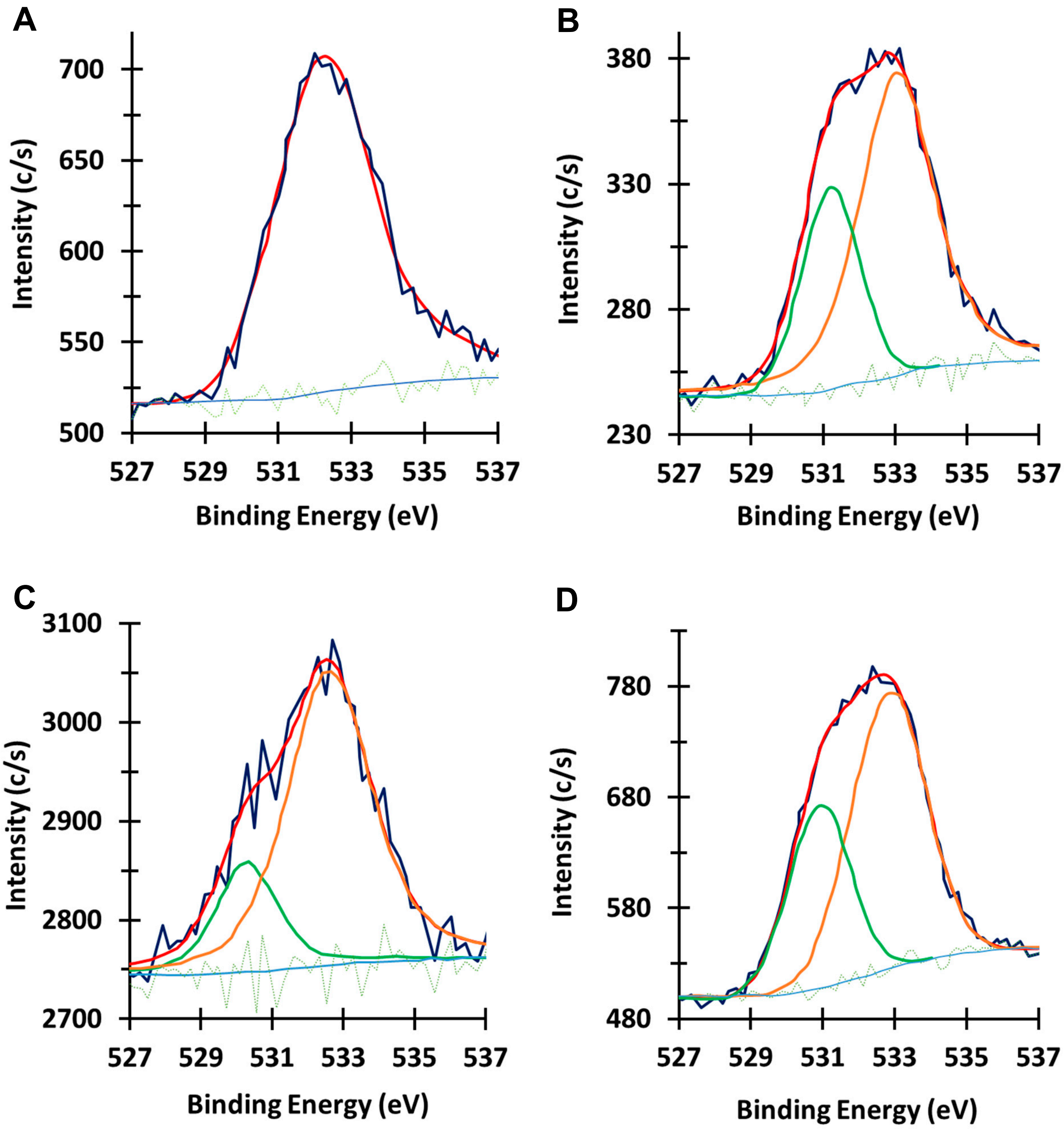


**Figure S13.** Representative O1s XPS spectra of (**A**) 2015-pMWNTs, **(B)** 2015-cMWNTs, **(C)** 2018-pMWNTs, and **(D)** 2018-cMWNTs. The dark-blue traces are the raw spectra and the red traces are the corresponding best fits; the light-green and light-blue traces are the raw background and smoothed background, respectively; and the green and orange traces are the best Gaussian fits.

**Table S2.** Analyses of the O1s XPS peaks from pMWNT and cMWNT powders.[a]

| MWNT Powder | Peak 1 Position (eV) | Peak 1 Area (%) | Peak 2 Position (eV) | Peak 2 Area (%) |
|---|---|---|---|---|
| **2015-pMWNTs** | 532.14 | 100 | ---- | ---- |
| **2015-cMWNTs** | 533.09 | 70 | 531.25 | 30 |
| **2018-pMWNTs** | 532.67 | 78 | 530.37 | 22 |
| **2018-cMWNTs** | 532.86 | 65 | 530.94 | 35 |

[a] Peak positions and areas determined from the O1s XPS peaks of the four MWNT powders shown in Figure S13. The O1s peak of the 2015-pMWNTs could be fit with a single Gaussian peak, while the O1s peaks of the other MWNT powders were best fit with two Gaussian peaks.
